# Frameworks to support evidence-informed decision-making in public health and infectious disease prevention and control: a scoping review

**DOI:** 10.2807/1560-7917.ES.2025.30.19.2400185

**Published:** 2025-05-15

**Authors:** Yang Song, Javier Bracchiglione, Jose F Meneses-Echávez, Helena de Carvalho Gomes, Barbara Albiger, Ivan Solà, David Rigau, Pablo Alonso-Coello

**Affiliations:** 1 School of Medicine, Chinese University of Hong Kong, Shenzhen, China; 2 Centro Cochrane Iberoamericano, Barcelona, Spain; 3 Institut de Recerca Sant Pau (IR SANT PAU), Barcelona, Spain; 4 Centro de Investigación Biomédica en Red de Epidemiología y Salud Pública, Instituto de Salud Carlos III, Madrid, Spain; 5 Interdisciplinary Centre for Health Studies (CIESAL), Universidad de Valparaíso, Viña del Mar, Chile; 6 Facultad de Cultura Física, Deporte, y Recreación, Universidad Santo Tomás, Bogotá, Colombia; 7 Norwegian Institute of Public Health, Oslo, Norway; 8 European Centre for Disease Prevention and Control (ECDC), Stockholm, Sweden

**Keywords:** infections, decision-making, health planning guidelines, public health, prevention and control

## Abstract

**Background:**

Evidence-informed public health decision-making (EIDM) is a complex process that must consider multiple factors.

**Aim:**

We aimed to identify and describe existing frameworks supporting evidence-informed public health decision-making and their application to infectious disease.

**Methods:**

We conducted a scoping review to describe current EIDM framework use in public health. We included decision-making frameworks in public health and examples of their use in infectious diseases. We searched MEDLINE and Health Systems Evidence from inception to December 2022. We also hand searched websites of relevant organisations and conducted a forward citation search of the included frameworks. Two reviewers selected studies independently, one reviewer extracted data and one cross-checked for accuracy. We presented the results narratively.

**Results:**

We included 15 frameworks. Seven had a generic scope and eight were focused on specific topics (immunisation, COVID-19 or other, non-infectious diseases). From the included frameworks, we identified a total of 18 criteria with each framework assessing a median of eight, the most frequent being ‘desirable effects’, ‘resources considerations’ and ‘feasibility’. We identified infectious disease examples for four frameworks: ‘Grading of Recommendations, Assessment, Development, and Evaluation’ (GRADE), WHO-INTEGRATe Evidence (WHO-INTEGRATE), ‘Ethics, Equity, Feasibility, and Acceptability’ (EEFA) and ‘Community Preventive Services Task Force’ (CPSTF) evidence-to-decision frameworks.

**Conclusion:**

Although several EIDM frameworks exist for public health decision-making, most have not been widely applied to infectious diseases. Current EIDM frameworks inconsistently address factors for public health decision-making. Further application and evaluation, and possibly adaptation of existing frameworks, is required to optimise decision-making in public health and infectious diseases.

## Introduction

The process of formulating evidence-based recommendations and policies entails making decisions suitable for relevant stakeholders. However, whether in a public health or a clinical context, it is a complex process [1,2]. Evidence-informed decision-making (EIDM) processes need to explicitly consider the best available evidence from research, the context and relevant experience [2,3]. Nevertheless, the range of factors that play a role is vast, and they depend on the type of decision, the perspective and the decision-making context [4].

Evidence-informed decision-making frameworks enable users to consider criteria (we will refer to decision-making factors, domains and considerations as criteria), regardless of the final type of decision (e.g. clinical recommendations, health systems or public health decisions) [5,6]. A number of frameworks have been proposed to address this process, such as the ‘Grading of Recommendations Assessment, Development and Evaluation’ (GRADE) evidence-to-decision (EtD) framework [5], or the World Health Organization (WHO) ‘INTEGRATe Evidence’ (INTEGRATE) framework [7]. Some research groups and organisations have launched other frameworks and processes, often aligned with the criteria and subcriteria contained in the GRADE EtD framework [7,8]. Some approaches emphasise specific criteria, such as equity in the Guidance for Priority Setting in Health Care (GPS-Health) [9], or ethics as in the 'decision-making triangle' [10]. Despite some variation in terms of the criteria being proposed, these frameworks aim to offer a comprehensive list of criteria that need to be considered by both decision-makers and guideline developers to facilitate a transparent decision-making process.

Infectious diseases remain a leading cause of morbidity and mortality worldwide. While HIV, tuberculosis and malaria are still causing 10% of all deaths each year, new infectious diseases, such as COVID-19, are continuously emerging [11]. The characteristics of infectious diseases may generate particular needs for the EIDM process, i.e. considering other bodies of evidence, such as mathematical models to estimate disease transmission, or taking into consideration the social impact of measures, such as quarantines. Furthermore, recent examples underline the need and usefulness of frameworks to develop rapid recommendations amid public health emergencies, such as the evidence-based recommendations for the prevention of COVID-19 or management of patients with COVID-19, issued by organisations such as the WHO [12], the European Centre for Disease Prevention and Control (ECDC) [13], the Infectious Disease Society of America (IDSA) [14] or the Association of the Scientific Medical Societies (AWMF) in Germany [15].

Previous reviews have described the available frameworks for different types of decisions, including healthcare coverage decisions [16] or environmental health interventions [17]. However, to the best of our knowledge, there has not been a systematic evaluation in the field of public health or in the field of infectious disease prevention and control. Therefore, the present review aims to identify, summarise and compare EIDM frameworks in public health, identifying common criteria to support timely and transparent decision-making, and clarify their application to infectious disease prevention and control.

## Methods

We conducted a scoping review (ScR) to address four research questions (RQ): (i) which frameworks have been proposed in public health for moving from evidence to decisions, recommendations, and/or policy? (RQ1), (ii) which criteria or domains are included in the frameworks identified in RQ1? (RQ2), (iii) how have these frameworks been used for public health decision-making in the field of infectious disease prevention and control? (RQ3), and (iv) what is the experience of using these frameworks? Which enablers and limitations have been identified for the implementation of the frameworks from experiences in RQ3? (RQ4).

The protocol for this research was previously published on Open Science Framework [18]. In this manuscript, we describe the results for RQ1, RQ2 and RQ3. Considering the length and volume of the study results, the findings for RQ4 are published separately [19,20]. We followed the Preferred Reporting Items for Systematic Reviews and Meta-Analysis (PRISMA) extension for ScR for reporting our research [21].

### Eligibility criteria

For RQ1 and RQ2, we included documents describing a formal EIDM framework in public health, defined as a structured process (explicitly describing or detailing domains, factors or criteria considered) supporting panels or stakeholders to move from the available evidence to a recommendation or decision that can potentially affect groups of people or that entire population [1,22]. For RQ3, we included examples of the implementation of public health EtD frameworks in the field of infectious disease prevention and control. We included documents published in English from the past 10 years (2013–2022).

### Information sources and search strategy

We systematically searched MEDLINE (via PubMed) and Health Systems Evidence from inception to December 2022 using search terms ‘decision making’, ‘evidence’, ‘evidence-based medicine’ and ‘health policy’. The full search strategy is appended in Supplement S1. We submitted the complete search strategy to peer review according to the Peer Review of Electronic Search Strategies (PRESS) checklist [23]. We also hand searched 60 websites of public health organisations and relevant scientific societies worldwide suggested by ECDC (the full list of stakeholder websites is available in Supplement S2), based on their expertise and knowledge of the public health field with a focus on infectious disease prevention and control. To retrieve unpublished material, we directly contacted key public health organisations via email and asked pre-designed questions regarding further EIDM frameworks that the organisations have used and for any examples using the identified frameworks. Additionally, we forward-tracked citations from the identified frameworks to retrieve examples of their use and other potential frameworks using Google Scholar ( https://scholar.google.com/) in May 2023.

### Data management and selection process

After deduplicating the records retrieved from the electronic databases in EndNote X20, we exported the unique references to Covidence (https://www.covidence.org/) for eligibility assessment. Two reviewers (JB and YS) conducted the screening process independently, first by title and abstract and then by full text. Discrepancies were solved by consensus, or by a third reviewer (either DR or PA-C). After completing the selection process from the database searches, one author (JB) hand searched websites and performed the forward citation search in Google Scholar. A second author (YS, DR or PA-C) cross-checked the findings.

### Data collection and data items

We designed a data extraction form based on our pilot testing with three identified frameworks and calibrated reviewers for data extraction. One reviewer (YS, JM-E) extracted relevant data from all the frameworks and a second reviewer (JB, YS, JM-E) verified the quality of the data. For RQ1 and RQ2, we obtained data about the framework's development organisation, scope (generic without topic-specific or topic-specific), target audience and settings, categories of decisions, methods for development, sources of evidence to inform these criteria and funding sources. For RQ3, two authors (YS, JB) selected a purposive sample of examples about the use of the frameworks, prioritising those that (i) involved a public health decision, (ii) were applied to the field of infectious diseases control and prevention, and (iii) were conducted in European countries (if available). We defined examples as fulfilling the eligibility criteria. Two authors (YS, JM-E) extracted the following data: the scope of the application, organisations, panel member profiles, methodology of the framework applications, technic used for reaching a final decision and tailoring of the frameworks.

### Data synthesis

We performed a descriptive analysis of the characteristics of each identified framework and example. For RQ1 and RQ2, we compared and described common characteristics, domains, criteria or features from the included frameworks and commented on differences and their explanation or rationale. To describe the decision-making criteria, we calculated the absolute frequencies and proportions of each criterion included in the identified frameworks. For RQ3, we grouped the described experiences according to the variables extracted during the data collection process. We presented the study findings in tabular format and reported narratively.

## Results

### Search results

We identified 3,892 citations from the literature search. After eliminating 26 duplications, we screened 3,866 for title and abstract and 204 for full-text assessment. We further excluded 162 documents due to the following reasons: not an EtD framework (n = 119), non-public health decision (n = 18), language other than English (n = 14), does not describe domains, factors or criteria considered for decision-making (n = 8), non-structured process (n = 2), published before 2013 (n = 1). This resulted in 26 publications describing 14 unique EIDM frameworks in public health [1,5,7,24-46] and 12 references describing experiences using the frameworks [25,47-54] from the literature search. Supplement S3 lists the studies excluded at this stage.

Through a website hand search, we retrieved five additional documents complementing the description of the identified frameworks [55-59] and one new framework [60]. We also contacted the 60 hand-searched organisations in Supplement S2 for additional frameworks or potential examples of identified frameworks. Thirteen organisations responded and no new eligible framework was identified. One example of the included EIDM frameworks in public health was provided by the responders [60], and subsequently confirmed by two authors (JB and YS). In total, we identified seven references through the website search.

From the set of 15 included frameworks, we selected the main references and performed a forward citation search using Google Scholar in May 2023. We screened a total of 655 references at this stage and retrieved one additional publication, complementing the description of an already identified framework [59]. For RQ3, we identified a purposive sample of 10 examples to illustrate the application of identified frameworks in the infectious disease field [61-70], all retrieved from the above-mentioned searches.

Finally, we included 15 unique decision-making frameworks corresponding to 33 publications for RQ1 and RQ2, 10 examples for RQ3 and 12 experiences of using the frameworks for RQ4. The selection process is summarised in the Figure. Supplement S4 provides a reference map for each RQ.

**Figure:**
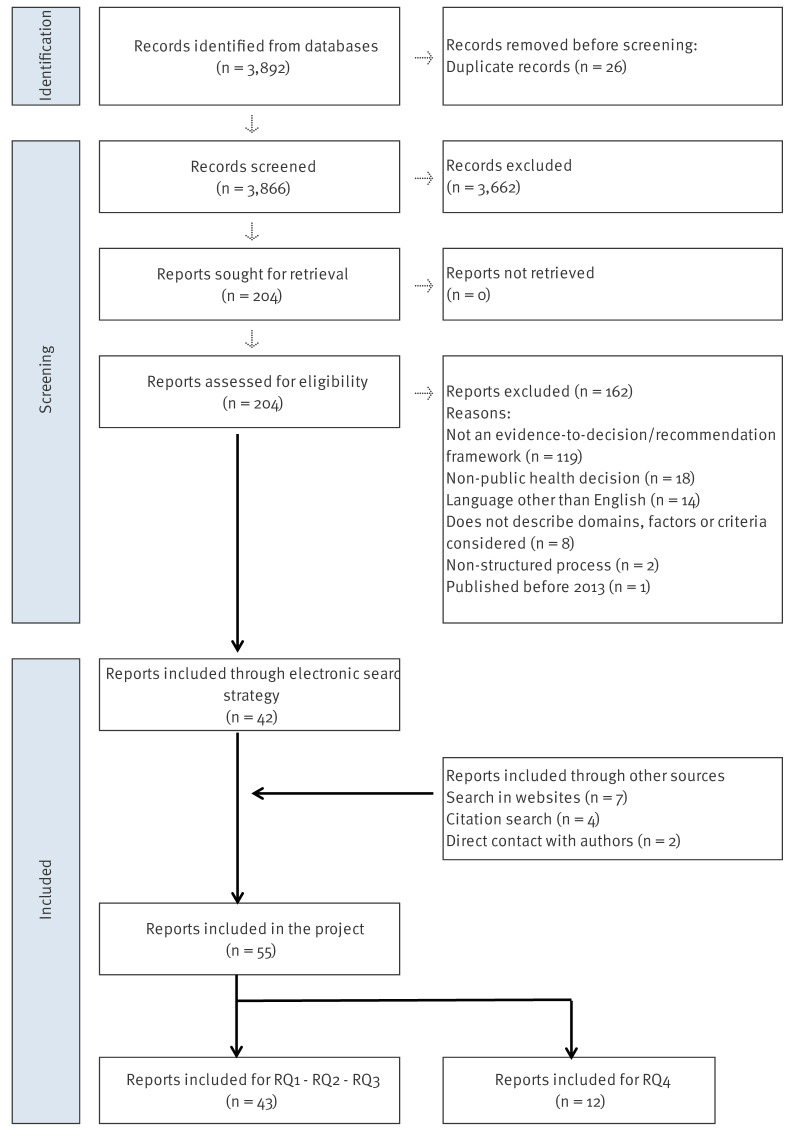
PRISMA flowchart summarising the selection process

RQ: research question.

RQ1: Which frameworks have been proposed in public health for moving from evidence to decisions, recommendations, and/or policy?

RQ2: Which criteria or domains are included in the frameworks identified in RQ1?

RQ3: How have these frameworks been used for public health decision-making in the field of infectious disease prevention and control?

RQ4: What is the experience of using these frameworks? Which enablers and limitations have been identified for the implementation of the frameworks from experiences in RQ3?

### Evidence-informed decision-making frameworks in public health

We identified 15 frameworks in public health decision-making (Table 1, Supplement S5). Of the 15 included EtD frameworks suitable for making a public health decision [1,7,26,28,30,32-39,60,71], seven frameworks were developed for a generic scope (that is, were developed to be applied in a broad range of health fields) [1,7,32-34,37,60], while eight were topic-specific, including immunisation programs [26], non-pharmacological interventions for COVID-19 [71], non-drug health technologies [36] or non-communicable diseases [28,30,35,38,39]. Two of the frameworks were developed by international organisations (GRADE working group [1] and WHO [7]), while others were from national [32,60] or institutional programmes of different countries (i.e. Australia [39], Canada [26,36-38], European Union [28], Germany [71], Iran [34], Nepal [33], United Kingdom [30] and United States (US) [35]).

**Table 1:** Characteristics of included evidence-to-decision frameworks (n = 15)

Framework name, publication year	Development organisation, region/country	Scope	Target audience	Targeted setting	Categories of decisions	Evidence used	Number of criteria^a^
**GRADE EtD framework, 2016** [1]	GRADE working group, international	Generic	Clinicians, guideline developers and policymakers	Not reported	Strength of the recommendation: strong or weak. Direction of the recommendation: for or against	Systematic reviews or research evidence developed using explicit methods	12
**WHO-INTEGRATE framework, 2019** [7]	WHO, international	Generic	Not reported	Applicable to all health interventions but particularly well suited for decisions about population-level and system-level interventions at both national and global levels	Not reported	Primary research, systematic reviews (formal evidence synthesis), or a more pragmatic approach (e.g. rapid reviews, umbrella reviews, formal consultation with experts - colloquial evidence)	6
**PSE framework, 2018** [35]	Division of Health Management and Policy, Institute of Public Health, Georgia State University, US	Topic specific (obesity prevention for local and national contexts)	Clinicians and collaborative groups	Local and state settings	Not reported	Not reported	3
**Framework for planning and improving evidence-based practices, 2019** [32]	CDC, US	Generic	Researchers, evaluators, health practitioners, funders and other decision-makers	US	Not reported	Preferably systematic reviews	2
**EEFA Framework, 2017** [26]	NACI, Canada	Topic specific (evidence-informed immunisation programme recommendations)	Advisory bodies in charge of implementing vaccine recommendations	Vaccine development within immunisation programs	Not reported	Not reported	4
**Framework for prioritising policy choices, 2013** [33]	The Resilient Mountain Solutions Initiative at ICIMOD, Nepal (supported by the Governments of Sweden, Norway and Regional Member Countries)	Generic	Policymakers and governments, as well as those interested in implementation	Global	Not reported	Not reported	13
**WICID framework, 2020** [71]	German government; Institute for Medical Informatics, Biometry and Epidemiology; and PSPHLMU, Bavaria, Germany	Topic specific (non-pharmacological interventions for COVID-19)	Decision-makers at the local, regional and national level	Local, regional and national levels	Not reported	Primary research, systematic reviews (formal evidence synthesis), or a more pragmatic approach (e.g. rapid reviews, umbrella reviews, formal consultation with experts - colloquial evidence)	11
**EURRECA framework, 2013** [28]	EURRECA network of excellence, European Union	Topic specific (micronutrient recommendations)	Public health policymakers	Not reported	Not reported	Nutritional and epidemiological science, evidence on the distribution of usual intake from monitoring surveys, evidence on consumer behaviour and social sciences, as well as stakeholder expertise	4
**Ontario Decision Framework, 2018** [36]	Ontario Health Technology Advisory Committee, Canada	Topic specific (nondrug health technologies)	Not reported	Single provincial portal for recommendations on the introduction of nondrug health technologies	Not reported	Scoping reviews, qualitative research synthesis, research synthesis related to health equity, ethics studies and patient preferences	3
**Policy Framework for Technology Assessment, 2018** [37]	The Technology Assessment Unit, McGill University Health Centre, Canada	Generic	Hospital administrators	Hospital-based health technology assessment units	Approved, approved for evaluation, not approved	Not reported	7
**Policy Framework for Primary Prevention of Occupational Cancer, 2017** [38]	Occupational Cancer Research Centre, Cancer Care Ontario, Canada	Topic specific (primary prevention of occupational cancer)	Not specified (various users and contexts)	Canada and other countries	Not reported	Jurisdictional evidence (no further details given)	5
**EVITA framework, 2020** [30]	Centre for Global Mental Health, Health Service and Population Research Department, Institute of Psychiatry, Psychology and Neuroscience, King’s College London, UK	Topic specific (mental health policy agenda)	Researchers, individuals and organisations working in mental health research policy ecosystem, such as policymakers, health policy agencies and planners	Low- and middle-income countries	Not reported	Scientific evidence, implementation science/knowledge translation, academic public and policy engagement (such as universities’ policy outreach centres), additional non-research evidence	8
**Framework of evidence-based decision-making in health system management, 2022** [34]	Shiraz University of Medical Sciences, Iran	Generic, closer to health system management	Not reported	Global, but with a focus on low- and middle-income countries and limited resource settings	Not reported	Not reported	4
**PREVIDE framework, 2022** [39]	University of Queensland, Australia	Topic specific (Noncommunicable Disease Prevention, NCD)	Not reported	Clinical and public health organisations	Investment of time, resources, money and/or organisational inertia. No action (neutral position). Disinvestment	Traditional and non-traditional sources of evidence (e.g. innovation, experience)	4
**CPSTF framework, 2016** [60]	CDC, US	Generic	Not reported	High income countries	Recommend, with strong or sufficient evidence. Recommend against, with strong or sufficient evidence when the harms are greater than the benefits. Insufficient evidence when there is not enough evidence to determine intervention effectiveness or inconsistent evidence.	All types of comparative study designs (e.g. experimental studies with allocated control groups, observational studies with concurrent or historical control groups and observational studies with single group before-after comparisons of change)	6

CDC: Centers for Disease Control and Prevention; CPSTF: Community Preventive Services Task Force; EEFA: Ethics, Equity, Feasibility, and Acceptability; EtD: evidence-to-decision; EURRECA: EURopean micronutrient RECommendations Aligned; EVITA: EVIdence To Agenda; GRADE: Grading of Recommendations, Assessment, Development, and Evaluation; ICIMOD: International Centre for Integrated Mountain Development; INTEGRATE: INTEGRATe Evidence; NACI: National Advisory Committee on Immunization; NCD: Noncommunicable disease; PREVIDE: PREVention decIDE; PSE: Policy, Systems, and Environmental; PSPHLMU: Pettenkofer School of Public Health Munich; Germany; UK: United Kingdom; US: United States; WHO: World Health Organization; WICID: WHO-INTEGRATE COVID-19.

^a^ Criteria may include subcriteria. For more details, see Supplement S6.

Ten frameworks reported their target audiences, including relevant stakeholders, as clinicians [1,35], guideline developers [1], policymakers [1,28,30,33,71], health practitioners [32], funders [32] and hospital administrators [26,37]. Thirteen frameworks reported their targeted settings, addressing global or national settings to regional or local settings, applying to populational level, system levels or hospital-based levels and designed for high-income countries or low- and middle-income countries. Four frameworks explicitly reported categories of decisions (e.g. strength or direction of the recommendations, or approved or not approved for evaluation etc.) [1,37,39,60]. Most of the frameworks were developed based on either literature reviews or systematic reviews of published frameworks or used a specific framework such as GRADE EtD framework or WHO-INTEGRATE as a starting point, engaging stakeholders or experts’ inputs, with or without validation, in a real-life context. Funding resources were mostly from public funding agencies, such as universities and national or international funding from governments or research institutes. Table 1 summarises the main characteristics of the included frameworks. Supplement S5 provides further details on the frameworks.

### Evidence-informed decision-making framework criteria

In total, there were 18 criteria addressed by identified EIDM frameworks. The median number of criteria addressed by the included EIDM frameworks was 8, ranging from 2 to 13. Five frameworks covered at least half of the identified criteria (> 9 criteria), including frameworks for prioritising policy choices, GRADE EtD frameworks with a generic scope, Ontario Decision framework, WHO-INTEGRATE, and WHO-INTEGRATE COVID-19 (WICID) with a topic-specific scope. Nine EtD frameworks covered at least nine identified criteria, while the other six EtD frameworks covered seven or fewer criteria (Table 2).

**Table 2:** Criteria addressed by evidence-informed decision-making frameworks

Domains / factors (n = 18)	Number of frameworks including the domain / factor	EIDM frameworks (n = 15)	Number of criteria included in the framework
Problem priority	9	**Generic frameworks (n = 7)**	
Desirable effects	13	CPSTF framework^a^ [60]	7
Undesirable effects	10	Framework for planning and improving evidence-based practices [32]	9
Certainty of evidence (effects)	9	Framework for prioritising policy choices [33]	11
Balance of effects	9	Framework of EIDM in health system management [34]	4
Values	6	GRADE EtD framework^a^ [1]	13
Certainty of evidence (values)	2	Policy Framework for Technology Assessment [37]	9
Resources considerations	12	WHO-INTEGRATE^a^ [7]	17
Certainty of evidence (resources)	2	**Topic specific frameworks (n = 8)**	
Cost-effectiveness	8	EEFA framework^a^ [26]	6
Equity	10	EURRECA [28]	9
Acceptability	8	EVITA framework [30]	7
Feasibility	13	Ontario Decision Framework [36]	11
Autonomy	2	Policy Framework for Primary Prevention of Occupational Cancer [38]	6
Sustainability	4	Policy, Systems, and Environmental Approaches for Obesity Prevention [35]	9
Legal and regulatory considerations	5	PREVIDE [39]	7
Political considerations	6	WICID [71]	14
Human rights	3		

CPSTF: Community Preventive Services Task Force; EEFA: Ethics, Equity, Feasibility, and Acceptability; EIDM: evidence-informed decision-making; EtD: evidence-to-decision; EURRECA: EURopean micronutrient RECommendations Aligned; EVITA: EVIdence To Agenda setting; GRADE: Grading of Recommendations, Assessment, Development, and Evaluation; INTEGRATE: INTEGRATe Evidence; PREVIDE: A Qualitative Study to Develop a Decision-Making Framework (PREVention decIDE); WHO: World Health Organization; WICID: WHO-INTEGRATE COVID-19.

^a^ EIDM frameworks with available infectious diseases examples. The number of criteria include both main headings and subheadings.

Most of the frameworks (12–13 of 15) considered, among their criteria, assessments related to ‘desirable effects’, ‘resources considerations’ and ‘feasibility’. To a lesser extent, eight of 15 frameworks included ‘cost-effectiveness’ and ‘acceptability’ as criteria [26,33-35,37]. Some of the frameworks also considered additional factors for public health decision-making, including evidence gaps (n = 1); stakeholder engagement (n = 3); transferability (n = 1); complementarities and interactions among strategies (n = 1); social impact (n = 1); and implications for the course of the COVID-19 pandemic and its impact on health (n = 1).

Six frameworks specified the importance of each decision-making criterion, with some details on how to consider each criterion [1,7,26,28,60,71]. Two frameworks also emphasised the prioritisation of important criteria based on the views of stakeholders [7,37], while one framework provided different thresholds for assessing each criterion without further guidance on how to make a final decision [36]. Other frameworks did not provide any details on the final recommendation or decision process. Table 2 outlines the criteria considered by identified EtD frameworks, Supplement S6 presents the map of each criterion.

### The application of included evidence-to-decision frameworks

Up to December 2022, our forward citation search strategy identified these cited generic frameworks: GRADE EtD framework (published in 2016, 204 citations); WHO-INTEGRATE (published in 2019, 132 citations); the ‘Framework for planning and improving evidence-based practices’ (published in 2019, 85 citations); the ‘Framework for prioritising policy choices’ (published in 2013, 25 citations). However, for topic-specific frameworks, only two were cited more than 50 times: the ‘Policy, Systems, and Environmental (PSE) Approaches for Obesity Prevention’ framework (published in 2018, 88 citations) and the ‘Ethics, Equity, Feasibility, and Acceptability’ (EEFA) framework (published in 2017, 51 citations). The rest of the frameworks had less than 20 citations (published from 2013 to 2022).

Among the examples identified, we purposively selected four examples for the GRADE EtD framework [61-64], two for WHO-INTEGRATE [65,66], two for the EEFA framework [67,68] and two for the Community Preventive Services Task Force (CSPTF) framework [69,70]. All the selected examples on the basis of purposive selection addressed public health decision-making related to the infectious disease field. Three examples for the GRADE EtD framework had a nationwide focus and were selected from Germany [64], Norway [62] and the US [61], while one was published by the WHO, with a global focus [63]. One example of the WHO-INTEGRATE framework was from Germany [66], and one was developed by the WHO [65]. The examples for the EEFA [67,68] and CSPTF [69,70] frameworks were from Canada and the US, respectively, with no European examples identified.

The guideline examples developed using the GRADE EtD framework covered antibiotics [61,64], COVID-19 [62] and malaria [63]. The examples that used WHO-INTEGRATE addressed sanitation and health [65] and COVID-19 [66]. The guidelines following the CPSTF framework covered HIV and vaccination [69,70] and the EEFA framework covered vaccination of herpes zoster [67] and COVID-19 booster [68]. Panel members’ profiles from the application of the four frameworks covered multiple roles for decision-making, including methodologists, healthcare specialists (e.g. clinicians, primary care physicians, etc.), intended end-users (e.g. clinicians, programme managers, etc.) and patients and other representatives. The methodology of those sample guidelines that followed each framework always included an evidence synthesis, used specific methods of rating systems for the certainty of the evidence, and made final decisions through a consensus or voting process. The reporting of the process was suboptimal to allow a full description of the methodology for two of the frameworks (the CPSTF framework and the EEFA framework), as the panel composition and decision-making process were barely specified. None of the frameworks were tailored and none of the examples described the implementation methodology of the frameworks. Table 3 provides a detailed description of the included examples.

**Table 3:** Examples of use of frameworks for public health evidence-informed decision-making in the infectious disease field

Title	Infectious disease health condition	Scope	Organisation	Panel members' profiles	Methodology	Decision-making process	Tailoring of the framework
**GRADE EtD framework** [61] **(n = 4)**							
Implementing an antibiotic stewardship programme: guidelines by the Infectious Diseases Society of America and the Society for Healthcare Epidemiology of America [61]	Yes, (antibiotic stewardship programme)	Treatment	IDSA	Multidisciplinary experts from IDSA, the Society for Healthcare Epidemiology of America, representatives from diverse geographic areas, paediatric and adult practitioners and a wide breadth of specialties representing major medical societies	Conducted a systematic literature review, grading the certainty of evidence according to IDSA Handbook on Clinical Practice Guideline Development (based on the GRADE methodology) [73] Conflicts of interests were addressed according to IDSA guidelines	Consensus^a^ development based on evidence	No^b^
COVID-19-EPIDEMIC: should individuals in the community without respiratory symptoms wear facemasks to reduce the spread of COVID-19?–a rapid review [62]	Yes (facemask to prevent COVID-19)	Prevention	NIPH	Mainly methodologists, no external panel members participated	Most of the EtD criteria were informed by high-quality SRs, although this information was not presented clearly in the reports. Some primary studies were also used as evidence base. The evidence included was based on a rapid systematic review. Additional data were collected from national surveillance. The panel focused primarily on the priority of the problem and the effects of the options. The resource criteria were considered, but the evidence base was limited	Consensus^a^	No
Strategies to enhance rational use of antibiotics in hospital: a guideline by the German Society for Infectious Diseases [64]	Yes (use of antibiotics in hospital)	Implementation, prevention, treatment, diagnosis, surveillance	DGI	No information available	The recommendations were derived by consensus by the GDG based on review of the literature, taking into account relevance, evidence, applicability and practicability in German and Austrian acute-care hospitals	Consensus^a^	No information available
WHO guidelines for malaria [63]	Yes (malaria)	Prevention, treatment, diagnosis, surveillance	WHO	Membership included the following categories of stakeholders: • Relevant technical experts (e.g. clinicians with relevant expertise, epidemiologists, entomologists) • Intended end-users (programme managers and health professionals responsible for adopting, adapting and implementing the guidelines) • Patients and/or other representatives from malaria-endemic countries	The guideline was developed using the GRADE approach, for each EtD factor (desirable effects, undesirable effects, overall certainty of the evidence of effects, values, resource requirements, cost-effectiveness, equity, acceptability, feasibility, the guideline panel based on the systematic reviews).	Consensus^a^ and online voting: “the guideline development process aimed to generate group consensus^a^ through open and transparent discussion. In most cases, anonymous voting was used to judge the different criteria and develop the final recommendation in order to reduce peer pressure. Voting was used as a starting point to build consensus or to reach a final decision when no consensus was reached.”	No
**WHO-INTEGRATE (n = 2)**							
Guidelines on sanitation and health [65]	Yes (guidelines on sanitation and health)	Prevention, treatment, implementation	WHO	The GDG included 30 members with expertise across the various relevant content areas. The group was balanced in terms of gender and geography and included technical experts as well as end-users. The GDG also included a methodologist with experience in systematic reviews, the GRADE approach and translation of evidence into recommendations	Key methodological steps covered: 1. Formulating the scoping questions based on a robust conceptual framework 2. Prioritising key questions 3. Identifying and/or conducting systematic reviews to address the key questions 4. Assessing the quality of the evidence 5. Formulating recommendations and good practice actions 6. Writing the guidelines 7. Developing a plan for dissemination and implementation	Voting, consensus^a^	No
S3-guideline measures for the prevention and control of SARS-CoV-2 transmission in schools. Living Guideline [66]	Yes (S3-guideline measures for the prevention and control of SARS-CoV-2 transmission in schools. Living guideline)	Prevention and control	AWMF	Students, employees in the school sector (teachers, head teachers, special education teachers), parents, policymakers in school authorities, public health stakeholders (e.g. local health authorities, RKI), as well as scientific societies (various medical societies, educational societies)	Overall methodology included: (i) prioritisation of topics and key questions; (ii) systematic research and selection of evidence; (iii) critical appraisal of the evidence using GRADE for direct evidence of each question; (iv) development of the recommendations using the WHO-INTEGRATE framework; (v) structured consensus development	Structured consensus^a^ development: voting and consensus	No
**EEFA framework (n = 2)**							
An Advisory Committee Statement (ACS) National Advisory Committee on Immunization (NACI) - updated recommendations on the use of herpes zoster vaccines [67]	Yes (herpes zoster vaccines)	Prevention	NACI	Unclear, not well-reported. May include medical specialties, methodologists or health economy experts	In brief, the broad stages in the preparation of a NACI advisory committee statement are: (i) knowledge synthesis (retrieve and summarise individual studies, rank the level i.e. study design and quality of the evidence which are summarised in the Summary of Evidence Tables, (ii) synthesis of the body of evidence of benefits and harms, considering the quality of the evidence and magnitude of effects observed, (iii) translation of evidence into a recommendation	Voting. However, the EtD process is not well-reported	No
An Advisory Committee Statement (ACS) National Advisory Committee on Immunization (NACI) - guidance on COVID-19 vaccine booster doses: initial considerations for 2023 [68]	Yes (COVID-19 vaccine booster doses)	Prevention	NACI	Unclear, not well-reported. May include medical specialties, methodologists or health economy experts	On November 29, 2022, and December 13, 2022, the NACI COVID-19 working group and full NACI membership, respectively, reviewed the available evidence on epidemiology and vaccine protection, as well as planning considerations for the next steps of the COVID-19 booster programme, including ethics, equity, feasibility and acceptability considerations. NACI also recommended the continued application of the existing decision-making framework for booster doses	Voting. However, the EtD process is not well-reported	No
**Community Preventive Services Task Force (CPSTF) framework (n = 2)**							
HIV prevention: partner services interventions to increase HIV testing [69]	Yes (HIV)	Diagnosis, treatment	The Community Guide	NR	The guideline was developed based on SRs mainly regarding of effectiveness, applicability and generalisability issues, data quality issues, implementability, other benefits and harms, and cost. The GDG used their own standard to rate the certainty of evidence and strength of recommendations. The decision-making process was not reported in detail	NR	Unclear - no information to ascertain
CPSTF findings for increasing vaccination [70]	Yes (vaccination)	Intervention/treatment	The Community Guide	NR	The guideline was developed based on SRs mainly regarding effectiveness, applicability and generalisability issues, data quality issues, implementability, other benefits and harms, and cost. The GDG used their own standard to rate the certainty of evidence and strength of recommendations. The decision-making process was not reported in detail	NR	Unclear - no information to ascertain

ACS: Advisory Committee Statement; AWMF: Association of the Scientific Medical Societies in Germany; CPSTF: Community Preventive Services Task Force; DGI: German Society for Infectious Diseases; EEFA: ; EtD: Evidence to Decision; GDG: Guideline Development Group; GRADE: Grading of Recommendations, Assessment, Development, and Evaluation; HIV: Human immunodeficiency viruses; IDSA: Infectious Disease Society of America; INTEGRATE: INTEGRATe Evidence; NACI: National Advisory Committee on Immunization; NIPH: Norwegian Institute of Public Health; NR: not reported; RKI: ; SARS-CoV-2: severe acute respiratory syndrome coronavirus 2; SRs: ; WHO: World Health Organization.

^a^ Consensus means decision-making process based on general agreement.

^b^ The example states that recommendations were done according to IDSA guidelines, which do not have major modifications with respect to GRADE’s original proposal. However, in the guideline itself they do not describe the specific EtD process for each recommendation.

## Discussion

We identified 15 frameworks facilitating EIDM in public health, of which seven frameworks had a generic scope and two were infectious disease-oriented. The frameworks included a median of eight criteria, mostly related to desirable effects, resource considerations and the feasibility of the interventions. Some frameworks covered implementation considerations during the decision-making process to facilitate its future use. We identified examples of public health decisions applying to infectious disease control and prevention for four of the frameworks: GRADE EtD [1], WHO-INTEGRATE [7], EEFA [26] and CSPTF [60] frameworks. Despite selecting prioritised examples for each framework, most of the retrieved documents used the GRADE EtD framework, while WHO-INTEGRATE had fewer examples of use; however, this framework could be adopted by more institutions in the near future, as it is still recent. Both EEFA and CSPTF-selected examples were conducted and applied in North America, and we did not identify any European examples of their use.

Other reviews have assessed EtD frameworks for different types of decisions. Morgan et al. [16] mapped out 13 frameworks supporting healthcare coverage decisions using GRADE EtD criteria. They found that non-GRADE EtD framework criteria could be arranged inside the GRADE EtD criteria, with no criterion offering superior value to be considered as a standalone new criterion. In our review, specific criteria of some frameworks do not fully align with the domains of the GRADE EtD framework, such as the emphasised importance of legal and regulatory considerations and political considerations [30,38]. Norris et al. [17] reviewed existing EIDM frameworks for environmental health interventions and highlighted the lack of guidance for the decision-making process. Similarly, our study found that there is little information on common criteria definitions and less guidance about how to assess criteria, as several frameworks only briefly describe or name the considered criteria without providing further details, as shown in Supplement S5. This aligns with the limitations presented by Norris et al. [17] and precludes the development of a single standardised overarching framework.

Although public health decision-making frameworks and Health Technology Assessments (HTAs) have different focuses, they are closely linked and often overlap. Health Technology Assessments use systematic reviews and modelling to assess cost-effectiveness, societal impacts, ethical issues, and legal contexts, supporting decision-making across clinical and public health fields [72,73]. Similarly, public health frameworks evaluate desirable effects, resource needs, and feasibility. When resource considerations are key, HTAs provide valuable economic and contextual insights that enhance public health frameworks, making decision-making both evidence-based and resource-efficient.

Our study has several limitations. First, distinguishing between public health and clinical frameworks was challenging and some lacked detail. Second, our search was conducted until December 2022 and was limited to documents published in English. Although we cannot rule out the chance of having overlooked relevant frameworks, we applied a forward citation search in May 2023, and complemented it with a hand search of key organisations’ websites that allowed us to identify further studies relevant to our question. Third, some of the included frameworks may not be developed for the infectious disease field; however, we opted for inclusiveness to capture all characteristics that may be relevant. We did not systematically retrieve all examples for the selected frameworks. We rather searched for documents describing how each EtD framework had been used or tailored in a real-life context. Although we quantified the citations of each framework through our citation search strategy, we did not assess the proportion of self-citations. Therefore, the number of citations can only work as a proxy for the spread of frameworks through the community, rather than a global indicator of their use in practice. Last, the generalisability of our findings is limited by their focus on western European countries and the public health field. Additionally, our selection of examples may not fully represent cases identified or the broader range of potential decision-making scenarios.

This study was strengthened by an exhaustive and rigorous search, including database, web and citation searches. In addition, our study describes in detail how each framework facilitates EIDM processes, including an overall mapping of the criteria considered, and provides a comprehensive view of the EIDM process. We also conducted a systematic assessment of the application and experience of the EIDM frameworks, summarising the barriers and facilitators.

Our study provides a comprehensive review of the current EIDM frameworks in public health and their applications in a real-life context of infectious diseases. The included frameworks, which have been developed for or have been used within the public health field, are described in detail and can be used in the future to support decisions requiring frameworks. The most frequently addressed decision-making criteria were desirable effects, resources considerations and feasibility, emphasising their importance and suggesting these should be considered by future EtD processes in this field. In addition, to ensure that topics are relevant to target users and prioritised factors are addressed, stakeholder engagement was specifically mentioned as an important consideration for the EIDM process by three frameworks [30,35,38]. Involving decision related stakeholders can facilitate fair and comprehensive decisions with easier uptake [74-76]. A more comprehensive review of the application of current EIDM frameworks is needed in future studies to confirm the applicability of the identified frameworks and criteria.

## Conclusion

Although several EIDM frameworks exist for public health decision-making, most have not been widely applied in infectious diseases. Current EIDM frameworks inconsistently address the factors for public health decision-making. The identified heterogeneity of criteria included by each decision-making framework implies that there is room for improvement, but the balance between the comprehensiveness of a framework’s criteria and the efficiency of the EIDM process remains unknown. To enhance the transparency and accountability of decision-making in public health and infectious diseases, it is important to apply and test existing frameworks, evaluate their effectiveness and adapt them as needed to improve usability.

## Data Availability

All data are available upon request from the authors.

## Supplementary Data

670000 Supplementary Material
