# Supplementary material for: Frameworks to support evidence-informed decision-making in public health and infectious disease prevention and control: a scoping review
Source: Euro Surveill. 2025 May 15;30(19):2400185. doi: 10.2807/1560-7917.ES.2025.30.19.2400185 (PMC12083068; doi:10.2807/1560-7917.ES.2025.30.19.2400185)
Supplement: Supplementary Material [file 24-00185_CARVALHO_GOMES_Supplement.pdf]

This supplementary material is hosted by *Eurosurveillance* as supporting information alongside the article ‘Frameworks to support evidence-informed decision-making in public health and infectious disease prevention and control: a scoping review’, on behalf of the authors, who remain responsible for the accuracy and appropriateness of the content. The same standards for ethics, copyright, attributions and permissions as for the article apply. Supplements are not edited by *Eurosurveillance* and the journal is not responsible for the maintenance of any links or email addresses provided therein.

Supplement S1. Search strategy for MEDLINE/PubMed and Health Systems Evidence

**MEDLINE/PubMed**<br>***Date of last search: December 19, 2022***	
#1	"Decision Making"[Majr]
#2	evidence[ti]
#3	decision*[ti]
#4	recommendation*[ti]
#5	policy[ti]
#6	priority[ti]
#7	priorities[ti]
#8	prioritisation[ti]
#9	prioritization[ti]
#10	#1 OR #2 OR #3 OR #4 OR #5 OR #6 OR #7 OR #8 OR #9
#11	"Evidence-Based Medicine"[Mesh]
#12	"Health Policy"[Mesh]
#13	#11 OR #12
#14	approach*[tiab]
#15	formulat*[tiab]
#16	develop*[tiab]
#17	review*[tiab]
#18	synthes*[tiab]
#19	synthez*[tiab]
#20	guidance[tiab]
#21	criteria[tiab]
#22	methodolog*[tiab]

#23	inform*[tiab]
#24	guideline*[tiab]
#25	#13 OR #14 OR #15 OR #16 OR #17 OR #18 OR #19 OR #20 OR #21 OR #22 OR #23 OR #24
#26	framework*[ti]
#27	#10 AND #25 AND #26
#28	approach*[ti]
#29	formulat*[ti]
#30	develop*[ti]
#31	review*[ti]
#32	synthes*[ti]
#33	guideline*[ti]
#34	guidance[ti]
#35	criteria[ti]
#36	methodolog*[ti]
#37	inform*[ti]
#38	#13 OR #27 OR #28 OR #29 OR#30 OR #31 OR #32 OR #33 OR #34 OR #35 OR #36 OR #37
#39	theor*[ti]
#40	#10 AND #38 AND #39
#41	#27 OR #40

**Health Systems Evidence**<br>***Date of last search: December 19, 2022***
(evidence OR decision* OR recommendation* OR policy OR priority OR priorities OR prioritisation OR prioritization) AND (approach* OR formulat* OR develop* OR review* OR synthes* OR synthez* OR guidance OR criteria OR methodolog* OR inform* OR guideline* OR tool OR tools) AND (framework* OR theor*)

Supplement S2. List of relevant institutions and organisations for web search and surveys.

**Institution or organisation**	**Country**	**Website**
Federal Ministry of Health	Austria	http://www.bmg.gv.at/
Sciensano	Belgium	https://www.sciensano.be/en
National Centre of Infectious and Parasitic Diseases	Bulgaria	http://www.ncipd.org/
Croatian National Institute of Public Health	Croatia	http://www.hzjz.hr/epocetna.htm
Directorate of Medical and Public Health Services	Cyprus	http://www.moh.gov.cy/moh/moh.nsf/index_en/index_en
National Institute of Public Health (NIPH)	Czech Republic	http://www.szu.cz
Danish Health and Medicines Authority	Denmark	http://www.sundhedsstyrelsen.dk/English.aspx
Health Board	Estonia	http://www.terviseamet.ee
Finnish Institute for Health and Welfare	Finland	http://www.thl.fi
French Public Health Agency	France	http://www.santepubliquefrance.fr
Robert Koch Institute	Germany	http://www.rki.de
National Public Health Organization	Greece	https://eody.gov.gr/eody/
National Public Health Center, Ministry of Human Capacities	Hungary	https://2015-2019.kormany.hu/en/ministry-of-human-resources
Centre for Health Security and Communicable Disease Control, Directorate of Health	Iceland	http://www.landlaeknir.is/
Health Protection Surveillance Centre	Ireland	https://www.hpsc.ie/
Ministry of Health	Italy	http://www.salute.gov.it/
Centre for Disease Prevention and Control	Latvia	http://spkc.gov.lv/
Principality of Liechtenstein	Liechtenstein	http://www.ag.llv.li
Ministry of Health	Lithuania	http://www.sam.lt/

Health Directorate	Luxembourg	https://sante.public.lu/fr.html
Superintendence of Public Health	Malta	https://deputyprimeminister.gov.mt/en/sph/Pages/Superintendence-of-Public-Health.aspx
National Institute for Public Health and the Environment (RIVM)	Netherlands	http://www.rivm.nl/
Norwegian Institute of Public Health	Norway	http://www.fhi.no/
National Institute of Public Health/National Institute of Hygiene	Poland	http://www.pzh.gov.pl/
Directorate General of Health	Portugal	http://www.dgs.pt/
National Institute of Public Health	Romania	http://www.insp.gov.ro/
Public Health Authority of the Slovak Republic	Slovakia	http://www.uvzsr.sk/en/
National Institute of Public Health (NIJZ)	Slovenia	http://www.nijz.si
Ministry of Health, Social Services and Equality	Spain	http://www.msssi.es
Public Health Agency of Sweden	Sweden	https://www.folkhalsomyndigheten.se/
Africa Centres for Disease Control and Prevention (Africa CDC)	Africa	https://africacdc.org/
Australian Government Department of Health and Aged Care	Australia	https://www.health.gov.au/
Australian Commission on Safety and Quality in Health Care	Australia	https://www.safetyandquality.gov.au/
Ministério da Saúde Brazil	Brazil	https://www.gov.br/saude/pt-br
Public Health Agency of Canada	Canada	https://www.canada.ca/en/public-health.html
Canadian Task Force on Preventive Health Care	Canada	https://canadiantaskforce.ca/
Caribbean Public Health Agency	Caribbean	https://carpha.org/
Chinese Center for Disease Control and Prevention	China	https://www.chinacdc.cn/en/
Israel Ministry of Health	Israel	https://www.gov.il/en/departments/ministry_of_health/govil-landing-page
Japanese Ministry of Health, Labour and Welfare	Japan	https://www.mhlw.go.jp/english/

Korea Disease Control and Prevention Agency	Korea	https://www.kdca.go.kr/index.es?sid=a2
Mexico Ministry of Health	Mexico	https://www.gob.mx/salud
Singapore Ministry of Health	Singapore	https://www.moh.gov.sg/
Thailand Ministry of Health	Thailand	https://p4h.world/en/member/ministry-public-health-thailand
UK Health Security Agency	UK	https://www.gov.uk/government/organisations/uk-health-security-agency
Center for Disease Control and Prevention	USA	https://www.cdc.gov/
World Health Organization	International	https://www.who.int/
National Institute for Health and Care Excellence (NICE)	UK	https://www.nice.org.uk/
Health Information and Quality Authority	Ireland	https://www.hiqa.ie/
National Collaborating Centre for Methods and Tools	Canada	https://www.nccmt.ca/
Cochrane Public Health	International	https://ph.cochrane.org/
GRADE Working Group (public health group)	International	https://www.gradeworkinggroup.org/
Joanna Briggs Institute	International	https://jbi.global/
The Community Guide	-	https://www.thecommunityguide.org/
Joint Research Commission (JRC)	International	https://joint-research-centre.ec.europa.eu/index_en
European Commission	International (Europe)	https://commission.europa.eu/index_en
European Union (centres and agencies related to health)	International (Europe)	https://european-union.europa.eu/
International Network for Government Science Advice	-	https://ingsa.org/
Guideline International Network (GIN)	International	https://g-i-n.net/get-involved/resources
Infectious Disease Society of America (IDSA)	International	https://www.idsociety.org/

Supplement S3. Excluded studies by full-text screening, with reasons

Study ID	DOI	Reason for exclusion
Abbasian 2020	10.2147/RMHP.S258661	Not an EtD framework
Abbey 2017	10.1186/s12889-016-3957-1	Not an EtD framework
Aiassa 2022	10.14573/altex.2004211	Not an EtD framework
Akiyama 2021	10.1016/S2468-1253(20)30365-4	Not an EtD framework
Alonso-Coello 2018a	10.1016/j.gaceta.2017.03.008	Language other than English
Alonso-Coello 2018b	10.1016/j.gaceta.2017.02.010	Language other than English
Alsalem 2022	10.1007/s10462-021-10124-x	Not an EtD framework
AlSiyabi 2021a	10.1123/jpah.2021-0235	Not an EtD framework
AlSiyabi 2021b	10.1123/jpah.2021-0152	Not an EtD framework
Alva 2018	10.3390/ijerph15030522	Not an EtD framework
Ananthapavan 2021	10.1186/s12961-021-00796-w	Non-structured process
Angelis 2017	10.1016/j.socscimed.2017.06.024	Non-public health decision
Angelis 2020	10.1016/j.socscimed.2019.112595	Not an EtD framework
Association of Women's Health, Obstetric and Neonatal Nurses 2022a	10.1016/j.jogn.2022.01.001	Non-public health decision
Association of Women's Health, Obstetric and Neonatal Nurses 2022b	10.1016/j.nwh.2022.01.001	Non-public health decision
Baltussen 2017	10.1016/j.jval.2016.11.019	Does not describe domains, factors or criteria considered

Baltussen 2021	10.34172/ijhpm.2021.158	Does not describe domains, factors or criteria considered
Bao 2021	10.1186/s12913-021-06827-0	Non-public health decision
Behzadifar 2021	10.15167/2421-4248/jpmh2021.62.2.2041	Not an EtD framework
Benmarhnia 2017	10.15171/ijhpm.2017.28	Not an EtD framework
Bertone 2013	10.1186/1478-4505-11-39	Not an EtD framework
Blythe 2022	10.5334/ijic.5997	Not an EtD framework
Bowen 2016	10.2105/AJPH.2015.302970	Not an EtD framework
Brady 2016	10.1016/j.evalprogplan.2016.01.003	Not an EtD framework
Bragge 2017	10.1186/s12874-017-0314-8	Not an EtD framework
Brands 2018	10.3390/ijerph15050942	Not an EtD framework
Brindis 2014	10.1146/annurev-publhealth-032013-182455	Not an EtD framework
Brunton 2016	No DOI. https://eppi.ioe.ac.uk/CMS/Portals/0/PDF%20reviews%20and%20summaries/Employer-led%20workplace%20health%202016%20Brunton.pdf	Not an EtD framework
Backman 2022	10.34068/joe.60.02.21	Not an EtD framework
Caiaffa 2014	10.1007/s11524-013-9812-0	Not an EtD framework
Calonge 2022	10.1002/jrsm.1582	Not an EtD framework
Camps 2020	10.1200/JOP.19.00487	Not an EtD framework
Cao 2022	10.1111/jonm.13458	Not an EtD framework

Chambers 2015	10.1093/pubmed/fdu069	Not an EtD framework
Chan 2020	10.1136/bmjopen-2019-032884	Not an EtD framework
Ciro Correa 2020	10.1186/s12961-020-00588-8	Not an EtD framework
Cole 2015	10.5888/pcd12.150300	Not an EtD framework
Coles 2016	10.1111/1468-0009.12195	Not an EtD framework
Conrad 2019	10.1016/j.zefq.2019.02.006	Language other than English
Crépault 2016	10.1016/j.drugpo.2016.04.013	Not an EtD framework
Dahm 2017	10.1016/j.jclinepi.2017.02.019	Non-public health decision
Davies 2014	10.1016/j.puhe.2013.11.011	Not an EtD framework
De Pietro 2015	No DOI. PMID: 26766626	Not an EtD framework
deFolter 2018	10.1017/S0266462318000090	Non-public health decision
Dinda 2020	10.4103/ijmr.IJMR_3640_20	Not an EtD framework
Djulbegovic 2014	10.1200/JOP.2013.001364	Not an EtD framework
Dörr 2022	10.1371/journal.pone.0263898	Not an EtD framework
Escoffery 2018	10.1186/s13012-018-0815-9	Not an EtD framework
Field 2016	10.1186/s12961-016-0154-8	Not an EtD framework
Fischer 2021	10.1007/s11606-020-06451-4	Does not describe domains, factors or criteria considered
Fourn 2020	10.3917/spub.202.0273	Language other than English
FrutosPérez-Surio 2019	10.1186/s40545-019-0181-2	Not an EtD framework
Funk 2022	10.1016/j.healthpol.2021.10.001	Not an EtD framework
Gaffey 2021	10.1016/S0140-6736(21)00133-1	Not an EtD framework
Garcia 2018	10.5123/S1679-49742018000200020	Not an EtD framework

Gębska-Kuczerowska 2020	10.3390/ijerph17207657	Not an EtD framework
Glover 2020	10.1016/j.jclinepi.2020.06.004	Not an EtD framework
González-Lorenzo 2015	10.1016/j.vaccine.2014.12.020	Non-public health decision
González-Lorenzo 2016	10.1701/2152.23272	Language other than English
Grant 2022	10.1097/01.NUMA.0000874432.64403.fb	Not an EtD framework
Grill 2017	10.1007/s10728-015-0299-6	Not an EtD framework
Guo 2021	10.1186/s12875-021-01556-z	Not an EtD framework
Harder 2015	10.1016/j.healthpol.2015.02.010	Not an EtD framework
Harder 2017	10.2807/1560-7917.ES.2017.22.40.16-00620	Not an EtD framework
Hart 2022	10.2105/AJPH.2022.306929	Not an EtD framework
Hester 2022	10.1016/j.jpeds.2022.06.002	Not an EtD framework
Holly 2022	10.1002/lrh2.10295	Not an EtD framework
Inotai 2018	10.1080/14737167.2018.1508345	Not an EtD framework
IOM 2015	10.17226/19013	Does not describe domains, factors or criteria considered
Janati 2018	10.4314/ejhs.v28i3.8	Not an EtD framework
Jessani 2021	10.1186/s12961-021-00733-x	Not an EtD framework
JimenezdelaJara 2015	10.1186/0717-6287-48-10	Not an EtD framework
Jones 2017	10.1016/j.socscimed.2017.01.048	Not an EtD framework
Jones-Bonofiglio 2020	No DOI. PMID: 32880333	Not an EtD framework
Kallenbach 2019	10.1016/j.zefq.2019.06.001	Language other than English

KamphuisCBM 2022	10.1093/eurpub/ckac068	Not an EtD framework
Keygnaert 2016	No DOI. PMID: 27786434	Not an EtD framework
Kim 2019	10.1016/j.ypmed.2019.105781	Does not describe domains, factors or criteria considered
Kolasa 2018	10.1080/14737167.2018.1467759	Non-public health decision
Kuchenmüller 2022	10.1016/j.evalprogplan.2022.102053	Not an EtD framework
Kumar 2020	10.1093/heapol/czaa027	Not an EtD framework
Lalani 2018	10.1111/hex.12852	Not an EtD framework
Lane 2021	10.1108/LHS-03-2021-0013	Not an EtD framework
Lewin 2019	10.1186/s12961-019-0468-4	Not an EtD framework
Li 2017	10.12688/f1000research.10966.1	Not an EtD framework
Li 2022	10.1016/j.imr.2022.100841	Does not describe domains, factors or criteria considered
Lietz 2020	10.1016/j.zefq.2020.03.002	Language other than English
Lin 2020	10.1007/s11606-020-05783-5	Not an EtD framework
Lo 2019	10.1136/bmjopen-2018-026482	Not an EtD framework
Lotfi 2022	10.1016/j.jclinepi.2021.09.028	Not an EtD framework
Luoto 2013	10.1371/journal.pmed.1001469	Not an EtD framework
Mahdavi 2021	10.34172/ijhpm.2021.142	Not an EtD framework
Malin 2022	10.1007/s15010-021-01645-2	Non-public health decision
Maree 2021	10.1071/AH19290	Not an EtD framework
Martins 2021	10.1007/s11524-021-00560-z	Not an EtD framework
McLaren 2016	10.1002/14651858.CD010166.pub2	Not an EtD framework

McPhaul 2013	10.3912/OJIN.Vol18No01Man04	Not an EtD framework
Morche 2018	10.1016/j.zefq.2018.03.004	Language other than English
Morgan 2018	10.1016/j.jclinepi.2017.09.023	Non-public health decision
Morgano 2017	10.1701/2802.28354	Language other than English
Morgano 2018	10.1701/2902.29246	Language other than English
Mostafavi 2016	10.5539/gjhs.v8n10p212	Not an EtD framework
Murad 2020	10.1016/j.mayocp.2020.05.009	Not an EtD framework
Murphy 2021	10.3310/hta25760	Non-public health decision
Mwendera 2017	10.1186/s12961-017-0264-y	Not an EtD framework
Neale 2019	10.1093/advances/nmy113	Not an EtD framework
Neumann 2018	10.1016/j.jval.2017.12.012	Not an EtD framework
Nicod 2017	10.1007/s10198-016-0823-0	Non-public health decision
Norton 2019	10.1186/s12961-019-0463-9	Not an EtD framework
Nussbaumer-Streit 2018	10.1016/j.zefq.2018.05.004	Language other than English
Oxman 2010	10.1016/S0140-6736(09)61251-4	Published before 2013
Palazzo 2016	10.1701/2218.23926	Language other than English
Parmelli 2017	10.1017/S0266462317000447	Non-public health decision
Paulden 2015	10.1007/s40273-014-0235-x	Non-public health decision
Perfetto 2018	10.1016/j.jval.2017.12.002	Not an EtD framework
Pertschuk 2013	10.1097/PHH.0b013e3182582a57	Not an EtD framework
Pfeifflé 2019	10.3390/nu11020362	Not an EtD framework
Poulin 2013	10.2147/MDER.S51384	Not an EtD framework

Prasinos 2022	10.1109/JBHI.2022.3142503	Not an EtD framework
Qin 2020	10.1371/journal.pone.0237342	Non-public health decision
Quilodrán 2021	10.5867/medwave.2021.04.8182	Language other than English
Redman 2015	10.1016/j.socscimed.2015.05.009	Not an EtD framework
Restar 2019	10.1371/journal.pone.0224133	Not an EtD framework
Rodes-Sanchez 2022	10.1016/j.vaccine.2022.05.054	Not an EtD framework
Rütten 2016	10.1055/s-0035-1548883	Language other than English
Rycroft-Malone 2013	10.1186/1748-5908-8-28	Not an EtD framework
Sacks 2020	10.1007/s13679-020-00376-z	Not an EtD framework
Schloemer 2018	10.1186/s13012-018-0751-8	Not an EtD framework
Schoelles 2017	10.23970/AHRQEPCWHITEPAPER3	Not an EtD framework
Sculpher 2018	10.1016/j.jval.2017.12.003	Not an EtD framework
Shaban-Nejad 2017	10.3233/978-1-61499-830-3-1335	Non-structured process
Shah-Manek 2017	10.18553/jmcp.2017.23.6-a.s13	Non-public health decision
Shekelle 2013	No DOI. PMID: 23427349	Not an EtD framework
Silva 2016	10.1016/j.healthpol.2016.01.005	Not an EtD framework
Sin 2015	10.12809/hkmj144326	Not an EtD framework
Siu 2015	10.12809/hkmj144307	Not an EtD framework
Sofi-Mahmudi 2022	10.1093/heapro/daab049	Not an EtD framework
Solow 2018	10.1016/j.jval.2017.12.004	Not an EtD framework
Sosa 2021	10.1007/s10995-020-03018-x	Does not describe domains, factors or criteria considered
South 2019	10.1093/heapro/dax083	Not an EtD framework

Stafinski 2011	10.2165/11539840-000000000-00000	Non-public health decision
Tan 2019	10.1177/1355819619842305	Not an EtD framework
Thompson 2022	10.1186/s12961-022-00902-6	Not an EtD framework
Timotijevic 2013	10.1080/10408398.2012.747485	Does not describe domains, factors or criteria considered
Turner 2017	10.1001/jamapediatrics.2017.1360	Not an EtD framework
Unsworth 2021	10.1177/20552076211018617	Not an EtD framework
Vélez 2020	10.1186/s12961-020-00584-y	Not an EtD framework
Venkatesan 2019	10.1371/journal.pone.0223946	Not an EtD framework
Votruba 2021	10.1186/s12961-020-00651-4	Not an EtD framework
WaltersLEM 2018	10.2196/jmir.9940	Not an EtD framework
Weber 2017	10.1186/s12904-017-0252-6	Not an EtD framework
Wende 2022	10.1186/s43058-022-00316-z	Not an EtD framework
WHO 2013	No DOI. https://apps.who.int/iris/handle/10665/131300	Not an EtD framework
WHO 2018	No DOI. https://www.who.int/publications/i/item/9789241514088	Not an EtD framework
Wickremasinghe 2016	10.1093/heapol/czv079	Not an EtD framework
WongCHL 2020	10.1177/1534735420940418	Non-public health decision
Yazdi-Feyzabadi 2021	10.1186/s13690-021-00737-7	Not an EtD framework
Yearwood 2018	10.26633/RPSP.2018.91	Not an EtD framework
Yoder-Wise 2020	10.1111/nuf.12381	Not an EtD framework

Yue 2022	10.1186/s12913-022-07493-6	Not an EtD framework
Zawadzki 2021	10.1016/j.jval.2021.03.005	Not an EtD framework
Zucca 2021	10.3389/fpubh.2021.653588	Not an EtD framework

Supplement S4. List of included references and documents for each framework and research question

Framework	RQ1 - RQ2	RQ3	RQ4
**GRADE-EtD**	Main reference: [1]<br><br>Complementary references: [2–8]<br><br>Adaptations of the framework: [9–14]	[15–18]	[14,19–26]
**WHO-INTEGRATE**	Main reference: [27]<br><br>Complementary references: [28]	[29,30]	[31–33]
**PSE framework**	[34]	-	-
**Framework for planning and improving evidence-based practices**	[35]	-	-
**EEFA framework**	Main reference: [36]<br><br>Complementary references: [37,38]	[39,40]	-
**Framework for prioritising policy choices**	[41]	-	-
**WICID**	[42]	-	-
**EURRECA**	Main reference: [43]<br><br>Complementary references: [44]	-	-
**Ontario Decision Framework**	[45]	-	-
**Policy Framework for Technology Assessment**	[46]	-	-
**Policy Framework for Primary Prevention of Occupational Cancer**	[47]	-	-
**EVITA**	Main reference: [48]<br><br>Complementary references: [49]	-	-
**Framework of evidence-based decision-making in health system management**	[50]	-	-
**PREVIDE**	[51]	-	-

**CPSTF framework**	[52]	[53,54]	-

CPSTF: Community Preventive Services Task Force; EEFA: Ethics, Equity, Feasibility, and Acceptability; EtD: Evidence to Decision; EURRECA: EURopean micronutrient RECommendations Aligned; EVITA: EVIdence To Agenda; GRADE: Grading of Recommendations, Assessment, Development, and Evaluation; INTEGRATE: INTEGRATe Evidence; PREVIDE: PREVention decIDE; PSE: Policy, Systems, and Environmental; WHO: World Health Organization; WICID: WHO-INTEGRATE COVID-19.

Supplement S5. Detailed description of each identified framework

GRADE Evidence-to-Decision (EtD) framework [1]

Country	Multi-country
Development organisation	GRADE Working Group
Scope	Generic
Aim	To help groups of people (panels) use evidence in a structured and transparent way to inform decisions in the context of clinical recommendations, coverage decisions, and health system or public health recommendations and decisions
Target audience	Clinicians, guideline developers, and policymakers
Target setting	Not reported
Methods for development	Iterative process (i.e., literature review, brainstorming, stakeholder feedback, piloting, and user testing)
Funding source	European Commission FP7 Program (grant agreement 258583) as part of the DECIDE project
Conflict of interests	No financial relationships with any organisations that might have an interest in the submitted work in the previous three years, no other relationships or activities that could seem to have influenced the submitted work. Authors are members of the GRADE Working Group and the DECIDE project.
Categories for decisions	Strength of the recommendation: Strong or weak<br>Direction of the recommendation: For or against
Decision-making criteria	"For Health system and public health recommendations/decisions:<br>1. Priority of the problem<br>2. Desirable effects<br>3. Undesirable effects<br>4. Certainty of the evidence of effects<br>5. Values and preferences<br>6. Balance between desirable and undesirable effects<br>7. Resource requirements (costs)<br>8. Certainty of the evidence of resource requirements<br>9. Cost-effectiveness<br>10. Equity<br>11. Acceptability<br>12. Feasibility"
Description of the process to make the recommendations or decisions	"The panel reviews the judgments they have made for all of the criteria (assessment), considering the implications of those judgments for the recommendation or decision. The panel draws conclusions about the strength of the recommendation or type of decision. Besides, the panel states the recommendation or decision in a concise, clear, and actionable manner, along with the justification."
Definition of evidence in the framework	Research evidence refers to facts (actual or asserted) used to inform the panel's judgments that are derived from studies that used systematic and explicit methods
Specific type of evidence used	Systematic reviews or research evidence developed using explicit methods

WHO-INTEGRATE (INTEGRATe Evidence) framework [27]

Country	Global
Development organisation	World Health Organization
Scope	Generic
Aim	To ensure that all criteria of relevance in a given guideline or other health decision-making process are considered in a systematic way
Target audience	Not explicitly reported
Target setting	Applicable to all health interventions but particularly well suited for decisions about population-level and system-level interventions at both national and global levels
Methods for development	i) Analysis of WHO's norms and values; ii) systematic review of EtD criteria in clinical care and public health; iii) interviews with key informants (usefulness); iv) application to completed WHO guidelines; v) focus groups; vi) peer review; and vii) development of guidance and prompts for completing the EtD
Funding source	WHO Department of Maternal, Newborn, Child and Adolescent Health received grants from the United States Agency for International Development and the Norwegian Agency for Development
Conflict of interests	Two authors were members of the GRADE Working Group, and one author was a WHO employee
Categories for decisions	Not reported
Decision-making criteria	• "Balance of health benefits and harms<br>• Human rights and socio-cultural acceptability<br>• Health equity, equality, and non-discrimination<br>• Societal implications<br>• Financial and economic considerations<br>• Feasibility and health system considerations<br><br>The quality of evidence is considered a meta criterion across the above-mentioned six substantive criteria"
Description of the process to make the recommendations or decisions	"This must be an evidence-informed process. The framework is not intended to be a tick-box exercise, and must respond to a prioritisation of the most relevant criteria, subcriteria depending on the target question, and the time and resource disposition. All criteria are important and should be reflected on, but their relevance varies depending on the context. In contrast, not all subcriteria are always relevant"
Definition of evidence in the framework	Not reported
Specific type of evidence used	Primary research, systematic reviews (formal evidence synthesis), or a more pragmatic approach (e.g., rapid reviews, umbrella reviews, formal consultation with experts - colloquial evidences)

PSE-framework (Policy, Systems, and Environmental Approaches for Obesity Prevention: A Framework to Inform Local and State Action) [34]

Country	USA
Development organisation	Division of Health Management and Policy, Institute of Public Health, Georgia State University
Scope	Topic specific (obesity prevention for local and national contexts)
Aim	To provide guidance for clinicians and collaborative groups on the activities that hold promise for facilitating policy change for obesity prevention
Target audience	Clinicians and collaborative groups
Target setting	Local and state settings
Methods for development	Literature searches for published works that describe or explain the policymaking process
Funding source	Research grants from the US Centers for Disease Control and Prevention (CDC) and the Physical Activity Policy Research Network
Conflict of interests	Not reported
Categories for decisions	Promoting policy change by facilitating the convergence of the three domains.
Decision-making criteria	“Three domains: policy, systems, and environmental change<br>• The priority of the problem, acknowledged by policymakers<br>• The policy domain: identification of policy solutions targeting the problem (i.e., typically a specific determinant of obesity)<br>• The policy proposals that survive to ultimately receive serious consideration generally meet several selection criteria, including technical feasibility, congruence with values, and anticipation of future restraints (e.g., fiscal limitations, public acceptability, and politicians’ receptivity)<br>• The political domain represents the prevailing political context. This domain is affected by factors, such as national mood, public opinion, changes in administration, shifts in partisan or ideological distributions among politicians, and interest group pressure campaigns<br><br>Further, six activities are to be undertaken: (a) assess the social and political environment; (b) engage, educate, and collaborate with key stakeholders (public and political engagement); (c) identify and frame the problem; (d) utilise available evidence (evidence synthesis); (e) identify policy solutions, considering sustainability, effects on health equity, and any potential for unintended consequences); and (f) build support and political will”
Description of the process to make the recommendation or decision	Not reported
Definition of evidence in the framework	Not reported
Specific type of evidence used	Not reported

Framework for planning and improving evidence-based practices [35]

Country	USA
Development organisation	Center for Disease Control and Prevention (CDC)
Scope	Generic
Aim	• To promote dialogue among scientists and practitioners about a consistent taxonomy for classifying the evidence for public health practices<br>• To help researchers, practitioners, and evaluators show how their work contributes to building the evidence base for particular practices
Target audience	Researchers, evaluators, practitioners, funders, and other decision-makers
Target setting	USA
Methods for development	• Literature reviews of models and frameworks for classifying evidence, including best practices<br>• Mapping of "best practice" definitions and key criteria<br>• Deliberation among experts<br>• Development of a conceptual framework for planning and improving evidence-based practices by adapting and extending several streams of existing work related to developing a continuum of evidence<br>• Development of criteria, definitions, and examples for key terms and formulation of a series of questions to apply in assessing and classifying practices
Funding source	No funding
Conflict of interests	None to declare
Categories for decisions	Not reported
Decision-making criteria	“Two interrelated components: Public health impact (effectiveness, reach, feasibility, sustainability, and transferability) and Quality of evidence (ranging from weak to rigorous)”
Description of the process to make the recommendation or decision	Not reported
Definition of evidence in the framework	Not reported
Specific type of evidence used	Preferably systematic reviews

EEFA (Ethics, Equity, Feasibility, and Acceptability) Framework [36]

Country	Canada
Development organisation	The National Advisory Committee on Immunization (NACI)
Scope	Topic specific (evidence-informed immunisation program recommendations)
Aim	To systematically assess programmatic factors, such as the ethics, equity, feasibility, and acceptability of recommendations
Target audience	Advisory bodies in charge of implementing vaccine recommendations
Target setting	Vaccine development within immunisation programs
Methods for development	Five years of environmental scans, systematic reviews and surveys, refined by expert and stakeholder consultations and feedback
Funding source	Not reported
Conflict of interests	None to declare
Categories for decisions	Not reported
Decision-making criteria	• "Ethics integrated filters for content and process<br>a. Core ethical dimensions filter: Respect for persons and communities (informed choices); beneficence and non-maleficence; justice; trust<br>b. Ethical procedural considerations filter: Accountability; inclusiveness; responsibility; responsiveness; transparency<br>• Equity matrix: Pre-existing condition; place of residence; race/ethnicity/culture/ language/immigration/refugee status; occupation; gender identity/sex; religion/belief system; education/literacy level; socioeconomic status; social capital; age; other risk factors<br>• Feasibility matrix: Resources (vaccine supply, human resources, funding, and training); integration with existing programs (vaccine coverage, communication, co-admin with other vaccines and existing programs/schedules)<br>• Acceptability matrix: Vaccine (perceptions of); disease (perceptions of); process to get vaccinated; individual factors (beliefs, values, and experiences)"
Description of the process to make the recommendation or decision	• "Once the need for immunisation recommendations is identified, the Technical Leads use the evidence-informed tools to consider issues on ethics, equity, feasibility and acceptability and answer the specific questions from Erickson et al.'s Analytic Framework<br>• The Technical leads present the completed tools to the relevant NACI Working Group as part of the full evidence base considered when developing recommendations<br>• The conclusions of the EEFA Framework are presented within the spectrum of public health science for consideration by the jurisdictions in their own contexts, similar to a GRADE EtD table<br>• Links to the full EEFA Framework and supporting tools, as well as completed tools for the particular vaccine recommendations (if deemed necessary), will be attached to the NACI ACS"
Definition of evidence in the framework	Not reported
Specific type of evidence used	Not reported

Framework for prioritising policy choices [41]

Country	Nepal
Development organisation	The Resilient Mountain Solutions (RMS) Initiative at ICIMOD supported by the Governments of Sweden, Norway and Regional Member Countries.
Scope	Generic
Aim	To present principles and criteria, and a suggested approach for assessing and prioritising policy choices in planning and decision making
Target audience	Policy makers and governments, as well as those interested in implementation
Target setting	Global
Methods for development	Not reported
Funding source	Governments of Sweden, Norway and Regional Member Countries, and funds of ICIMOD contributed by the governments of Afghanistan, Norway, Sweden, Australia, Austria, Bangladesh, Bhutan, China, India, Myanmar, Nepal, Pakistan, and Switzerland
Conflict of interests	None to declare
Categories for decisions	Not reported
Decision-making criteria	• "Dimensions of priorities: saving human lives and livelihoods; efficiency and effectiveness; equity and fairness; sustainability and resilience<br>• Identifying smart strategies that bring synergistic effects<br>• Complementarities and interactions among strategies<br>• Assessing trade-offs, magnitude of the benefits<br>• Improving policy coherence<br>• Identify alternative approaches or combinations, weighing the potential benefits and externalities, both positive and negative, to maximise potential net benefits in achieving the broader societal goals<br>• Coherence, compatibility, and congruence<br>• Aligning Policy Instruments to Improve<br>• Policy Coherence<br>• Improving Policy Coherence<br>• Managing Externalities<br>• Reconciling Private and Social Interests<br>• Integrating Long-Term Sustainability in policy decisions "
Description of the process to make the recommendation or decision	Not reported
Definition of evidence in the framework	Not reported
Specific type of evidence used	Not reported

WICID (WHO-INTEGRATE COVID-19) [42]

Country	Global
Development organisation	German government; Institute for Medical Informatics, Biometry and Epidemiology (IBE); and Pettenkofer School of Public Health, LMU Munich, Bavaria, Germany
Scope	Generic
Aim	To support decision-makers in identifying and considering criteria of relevance for non-pharmacological interventions targeting COVID-19
Target audience	Those involved in making decisions on NPIs at the local, regional and national level (eg, from decision-makers deciding on municipal regulations of how to (re)open a specific school to decision-makers deciding on state-wide regulation on protective measures in the educational system), as well as the scientific expert groups advising these political decision-makers
Target setting	Local, regional, and national levels
Methods for development	• Authors employed the 'best fit' framework synthesis technique and used the WHO-INTEGRATE framework as a starting point<br>• Brainstorming<br>• A content analysis of twelve relevant documents intended to guide policymakers on the phasing out of applied lockdown measures in Germany<br>• Development of factors and criteria
Funding source	The authors received support from the Bundeszentrale für gesundheitliche Aufklärung (BZgA; the German Federal Center for Health Education) to cover the publication fees for this manuscript. The BZgA did not have any editorial or scientific influence on the content of this publication.
Conflict of interests	The first author is also the author of the WHO-INTEGRATE framework. Two authors were part of an expert group that developed strategy documents intended to inform the COVID-19 crisis task force of the German government. One (in the case of JMS) and two (in the case of MV) of which were included as comprehensive strategy documents in this analysis.
Categories for decisions	Not reported
Decision-making criteria	• "Implications for the course of the pandemic and its impact on health<br>• Implications for quality of life, social well-being and mental health<br>• Implications for physical health, health behaviour, health risks and healthcare beyond COVID-19<br>• Proportionality and accordance with individual autonomy and fundamental rights<br>• Acceptability of and willingness to implement the measures<br>• Equity, equality and the fair distribution of benefits and burdens<br>• Societal and environmental implications & considerations<br>• Economic implications & consideration<br>• Resource implications & considerations<br>• Feasibility implications & considerations<br>• Interaction with and implications for the health system<br><br>Quality of evidence considered as a meta criterion"
Description of the process to make the recommendation or decision	• "Development of a logic model or systems map of the measure and the context is intended to be implemented<br>• The WICID framework is used to expand on dimensions not adequately covered<br>• Identification of relevant stakeholders, informed by the logic model<br>• Those involved in the decision-making process need to define criteria that are assumed to be relevant for deliberating on the measure<br>• The assumed importance of the criteria should be rated (e.g., on a 1–5 scale from 'less important' to 'critical') and selected<br>• Efforts should be made to receive feedback on the expanded logic model and the selected criteria from key stakeholder groups identified

	in the mapping. Repeated rounds of steps 1–4 are likely to produce the best results<br>• Efforts should be made to acquire appropriate sources of evidence to inform the selected criteria (e.g., by commissioning research or inviting experts' judgments)<br>• The retrieved evidence for each criterion should be summarised and presented alongside the assessment of the quality of the evidence and its transferability to the context at hand<br>• The group of decision-makers should engage in the deliberation to balance the criteria against each other, taking their weight, direction, quality and transferability of the evidence into account<br>• The final judgement and the underlying rationale should be made transparent and public"
Definition of evidence in the framework	Not reported
Specific type of evidence used	Primary research, systematic reviews (formal evidence synthesis) or a more pragmatic approach (e.g., rapid reviews, umbrella reviews, formal consultation with experts - colloquial evidences-)

EURRECA (EURopean micronutrient RECommendations Aligned) [43]

Country	Europe
Development organisation	Eurreca network of excellence (NoE)
Scope	Topic specific (micronutrient recommendations)
Aim	To describe the process leading from assessing nutritional requirements to policy applications, based on evidence from science, stakeholders' interests, and the sociopolitical context. The framework also covers consumer issues and acknowledges the influences of the wider sociopolitical context
Target audience	Public health policy-makers
Target setting	Not reported
Methods for development	Review of conceptualizations on the process of setting micronutrient recommendations by three international organisations
Funding source	European Commission's Directorate General for Research
Conflict of interests	None to declare
Categories for decisions	Not reported
Decision-making criteria	• "Defining the nutrient requirements for health (aided by systematic reviews)<br>• Setting the nutrient recommendations<br>• Policy options<br>• Policy applications "
Description of the process to make the recommendation or decision	Not reported
Definition of evidence in the framework	Scientific evidence on health effects, biomedical factors, stage of life, susceptibility, geographical, socioeconomic cultural and religious factors
Specific type of evidence used	Nutritional and epidemiological science, evidence on the distribution of usual intake from monitoring surveys, evidence on consumer behaviour and social sciences, as well as stakeholder expertise

Ontario Decision Framework [45]

Country	Canada
Development organisation	Ontario Health Technology Advisory Committee (OHTAC)
Scope	Topic specific (nondrug health technologies)
Aim	To offer a transparent, multidisciplinary and consistent approach to making decisions in a deliberative manner
Target audience	Not reported
Target setting	Single provincial portal for recommendations on the introduction of nondrug health technologies
Methods for development	• A priori consensus on guiding principles<br>• A scoping review of decision attributes and processes used globally in health technology assessment (HTA)<br>• Presentations by methods experts and members of review committees<br>• Committee deliberations over a period of 3 years
Funding source	Not reported
Conflict of interests	Not reported
Categories for decisions	Not reported
Decision-making criteria	• "Context criteria<br>• Appraisal criteria: Benefits and harms, magnitude of certainty of evidence for benefits and harms, patients' perspectives, economics, summary of cost-effectiveness, patient-centred care (equity, solidarity, population health, collaboration, and shared responsibility for health)<br>• Feasibility criteria: Budget impact and organisational considerations"
Description of the process to make the recommendation or decision	"No general decision making process is reported. Different thresholds for assessing each criterion is provided, including a trigger tool to determine when a full ethics and social values analysis is warranted"
Definition of evidence in the framework	Not reported
Specific type of evidence used	Scoping reviews; qualitative research synthesis; research synthesis related to health equity, ethics studies, and patient preferences

Policy Framework for Technology Assessment [46]

Country	Canada
Development organisation	The Technology Assessment Unit (TAU) of the McGill University Health Centre (MUHC)
Scope	Generic
Aim	• To identify decision criteria specific to the context of hospital-based health technologies and interventions<br>• To estimate the extent to which the expert community agrees on the importance of the identified criteria<br>• To incorporate the identified criteria into a decision-aid tool<br>• To illustrate the application of a prototype decision-aid tool
Target audience	Hospital administrators
Target setting	Hospital-based health technology assessment (HTA) units
Methods for development	Relevant decision criteria were identified using existing frameworks for HTA recommendations, researchers past experience, literature search, and feedback from a survey of diverse stakeholders
Funding source	Not reported
Conflict of interests	None to declare
Categories for decisions	• Approved<br>• Approved for evaluation<br>• Not approved
Decision-making criteria	• "Clinical benefit: Magnitude of effectiveness, quality of evidence for effectiveness, and safety<br>• Impact on patient: Impact on patient convenience, patient preference, patient-centred outcome measures<br>• Value for money: Total cost, cost avoided/increased hospital efficiency, budget impact on other services, and cost-effectiveness<br>• Feasibility: Availability of local expertise, disruptiveness, need to generate local evidence, ability to increase cross-institution collaboration, personnel satisfaction<br>• Impact on healthcare system: Benefit to society (reduces health care costs), burden on other healthcare centres, and need (unnecessary duplication)<br>• Strategic considerations: Stakeholder pressure to acquire the technology; availability of external funding; number of patients affected by the technology<br>• Ethical considerations: Disruption of access to care"
Description of the process to make the recommendation or decision	"Not well-reported. The technical team documents research findings, indicating whether the findings for each criterion were favourable for the approval of routine use of ECMO in adults. The tool is then emailed to one member of the policy committee, who is asked to rate the importance of each criterion"
Definition of evidence in the framework	Not reported
Specific type of evidence used	Not reported

Policy Framework for Primary Prevention of Occupational Cancer [47]

Framework name	
Country	Canada
Development organisation	Occupational Cancer Research Centre, Cancer Care Ontario
Scope	Topic specific (primary prevention of occupational cancer)
Aim	To develop policies to prevent occupational cancer
Target audience	Not specified (various users and contexts)
Target setting	Canada and other countries
Methods for development	An environmental scan of existing prospective health policy analyses to identify potential parameters for a framework that can be used to develop occupational cancer primary prevention policies. The elements that routinely appeared in the literature and that were most applicable to occupational cancer primary prevention were ultimately chosen for inclusion.
Funding source	Canadian Cancer Society Research Institute (Grant #701285). There was no involvement of the funder in this manuscript
Conflict of interests	None to declare
Categories for decisions	Not reported
Decision-making criteria	• "Problem statement<br>• Context (structural, situational, cultural/social, and external factors)<br>• Jurisdictional evidence<br>• Primary prevention policy options<br>• Key policy players and their attributes"
Description of the process to make the recommendation or decision	Not reported
Definition of evidence in the framework	Evidence from other jurisdictions can be used to understand how a particular policy problem has been addressed elsewhere
Specific type of evidence used	Jurisdictional evidence (no further details given)

EVITA [48]

Framework name	
Country	Low and middle income countries
Development organisation	Centre for Global Mental Health, Health Service and Population Research Department, Institute of Psychiatry, Psychology & Neuroscience, King's College London
Scope	Topic specific (mental health policy agenda)
Aim	To facilitate, analyse and guide mental health research and policy interrelationships, with the intention to serve as a 'pragmatic, predictive, and effective tool' for improving research and policy exchange, and enhancing research impact on the policy agenda
Target audience	Researchers, individuals and organisations working in mental health research–policy ecosystem, such as policymakers, health policy agencies and planners
Target setting	Low and middle income countries
Methods for development	• Development of the provisional framework (EVITA 1.0)<br>• Validation framework for mental health<br>• Validation through in-depth interviews<br>• Revision and finalisation of the framework (EVITA 1.1)
Funding source	Not reported
Conflict of interests	Not reported
Categories for decisions	Not reported
Decision-making criteria	• "Advocacy coalitions: To achieve a uniform voice and policy ask, common ground of values, policy aims and implementation<br>• Engagement (actors): Stakeholder mapping (identification and recruitment)<br>• Evidence generators<br>• External influences (e.g., attitudes and perceptions of mental health, mental disorders, or the perception of psychology and psychiatry)<br>• Intermediaries: Their support role can expand into linking advocacy coalitions, to increase policy impact through their single vision and stronger political voice.<br>• Political context (policy making process, political will, motives and opportunities, and setting the political agenda)<br>• Mechanisms: capacity building; catalysts; communication/relationship/partnership building; strategic communication; building lasting relationships<br>• Framing: Identifying the status quo in relation to the issue, and in what way the new evidence diverges from this status quo, and then adapting the evidence to the context and policy question"
Description of the process to make the recommendation or decision	Not reported
Definition of evidence in the framework	The evidence has to be of good quality, rigorous, and trustworthy science, which is up to date, timely and relevant. Research (evidence) needs to be clear, understandable, and accessible to policy and public (open-access, and published in non-scientific media. It needs to be generalisable and applicable to local, regional or national policies
Specific type of evidence used	The evidence eco-system encompasses scientific evidence, implementation science/knowledge translation, and academic public and policy engagement (such as universities' policy outreach centres). It can be useful to consider additional non-research evidence

Framework of evidence-based decision-making in health system management [50]

Country of development	Iran
Development organisation	Shiraz University of Medical Sciences
Scope	Generic, closer to health system management (HSM)
Aim	To guide and adapt evidence-based decision-making in health system management
Target audience	Not reported
Target setting	Global, but with a focus on low and middle income countries and limited-resource settings
Methods for development	Systematic reviews, data analysis via thematic analysis, and concept generation to achieve the best-fit framework applying Carroll et al. 2013 approach
Funding source	Shiraz University of Medical Sciences, under code (96-01-07-14184)
Conflict of interests	Mrs. Tahereh Shafaghat conducted the project as part of the Ph.D. degree
Categories for decisions	Not reported
Decision-making criteria	"Four general phases of inquiring, inspecting, and implementing are integrated across 10 main steps<br>• Inquiring: 1) situation analysis and priority setting; 2) quantifying the issue and developing a statement; 3) capacity building and setting objectives; 4) evidence acquisition and integration<br>• Inspecting: 5) evidence appraisal; 6) Analysis, synthesis, and interpretation of data<br>• Implementing: 7) Developing Evidence-Based alternatives; 8) Pilot implementation of selected alternatives<br>• Integrating: 9) Evaluate alternatives; 10) Integrate and maintain change in practice "
Description of the process to make the recommendation or decision	Not reported
Definition of evidence in the framework	Not reported
Specific type of evidence used	Not reported

PREVIDE [51]

Country	Australia
Development organisation	University of Queensland, Australia
Scope	Topic specific (Noncommunicable Disease Prevention, NCD)
Aim	To develop a contemporary decision-making framework for NCD prevention in healthcare organisations
Target audience	Not reported
Target setting	Clinical and public health organisations
Methods for development	Qualitative study design (phenomenological), including cross-sectional and semi-structured interviews
Funding source	The University of Queensland Business School Connect Grant Scheme
Conflict of interests	None to declare
Categories for decisions	Investment of time, resources, money, and/or organisational inertia, no action (neutral position), or<br>disinvestment
Decision-making criteria	• "Data<br>• Evidence<br>• Ethics<br>• Health"
Description of the process to make the recommendation or decision	Not reported
Definition of evidence in the framework	Traditional and non-traditional sources of evidence (e.g., innovation, experience)
Specific type of evidence used	Not reported

Community Preventive Services Task Force (CPSTF) framework [55]

Country	USA
Development organisation	Center for Disease Control and Prevention, CDC
Scope	Generic
Aim	Not reported
Target audience	Not reported
Target setting	High income countries
Methods for development	Not reported
Funding source	Not reported
Conflict of interests	Not reported
Categories for decisions	• Recommend, with strong or sufficient evidence<br>• Recommend against, with strong or sufficient evidence when the harms are greater than the benefits<br>• Insufficient evidence, when there is not enough evidence to determine intervention effectiveness or inconsistent evidence
Decision-making criteria	• "Body of evidence (quality)<br>• Effectiveness<br>• Applicability<br>• Balance benef/harms<br>• Implementability<br>• Evidence gaps "
Description of the process to make the recommendation or decision	• "CPSTF decides on the topic for review based on their prioritisation process. From there, a coordination team ("the team") is convened to guide the review<br>• The team selects an intervention approach (a type of intervention that is used to address a specific public health problem, such as mass media campaigns to increase safety belt use) within the topic area for review<br>• Each team follows an extensive conceptualization process in which they draft a definition, inclusion and exclusion criteria, analytic framework, research questions, and applicability factors<br>• Next, the team consults with a research librarian at the CDC Library to draft a search strategy. The research librarian then conducts the systematic search<br>• Once candidate publications are obtained from the systematic search, the team begins a three-stage screening process to identify potential papers for inclusion<br>• The team narrows the search yield through the screening process and abstracts relevant information from the remaining papers using the Community Guide criteria to examine the quality of these papers<br>• The team analyses the data, calculating summary effect estimates and assessing applicability<br>• After completing the analysis, the team presents the findings to CPSTF, which translates evidence into CPSTF recommendations and broadly disseminates the findings to public health practitioners"
Definition of evidence in the framework	Not reported
Specific type of evidence used	All types of comparative study designs (e.g., experimental studies with allocated control groups, observational studies with concurrent or historical control groups, and observational studies with single group before-after comparisons of change)

Supplement S6. Map of EtD criterion

Domains/Criteria	CPSTF framework	EEFA framework	EURRECA	EVITA framework	Framework for planning and mproving evidence-based practices	Framework for prioritising policy choices	Framework of evidence-based decision-making in health system management	GRADE EtD framework	Ontario Decision Framework	Policy Framework for Primary Prevention of Occupational Cancer	Policy Framework for Technology Assessment	Policy, Systems, and Environmental Approaches for Obesity Prevention	PREVIDE	WHO-INTEGRATE	WICID
**Problem priority**	No	No	Yes	Yes	No	Yes	Yes	Yes	Yes	Yes	No	Yes	Yes	No	No
**Desirable effects**	Yes	Yes	No	Yes	Yes	Yes	Yes	Yes	Yes	Yes	Yes	Yes	No	Yes [1]	Yes
**Undesirable effects**	Yes	Yes	No	Yes	Yes	Yes	No	Yes	Yes	No	Yes	No	No	Yes [1]	Yes
**Certainty of the evidence of effects**	Yes	No	No	Yes	Yes	No	No	Yes	Yes	No	Yes	No	Yes	Yes	Yes
**Balance of effects**	Yes	Yes	Yes	No	No	Yes	Yes	Yes	Yes	No	No	No	No	Yes	Yes
**Values**	No	No	Yes	No	No	No	No	Yes	Yes	No	Yes	No	Yes	Yes [2]	No
**Certainty of evidence regarding values**	No	No	No	No	No	No	No	Yes	No	No	No	No	No	Yes	No
**Resources considerations**	No	No	Yes	Yes	Yes	Yes	Yes	Yes	Yes	No	Yes	Yes	Yes	Yes	Yes
**Certainty of evidence regarding resources**	No	No	No	No	No	No	No	Yes	No	No	No	No	No	Yes	No
**Cost-effectiveness**	No	No	Yes	No	Yes	Yes	No	Yes	Yes	No	Yes	No	No	Yes	Yes
**Equity**	No	Yes	Yes	No	Yes	Yes	No	Yes	Yes	No	No	Yes	Yes	Yes [2]	Yes
**Acceptability**	Yes	Yes	No	No	No	No	No	Yes	No	No	Yes	Yes	Yes	Yes	Yes
**Feasibility**	Yes	Yes	Yes	Yes	Yes	Yes [3]	No	Yes	Yes	Yes	Yes	No	Yes	Yes	Yes
**Autonomy**	No	No	No	No	No	No	No	No	No	No	No	No	No	Yes [4]	Yes
**Sustainability**	No	No	No	No	Yes	Yes	No	No	No	No	No	Yes	No	Yes[5]	No

**Legal and regulatory considerations**	No	No	Yes	No	No	No	No	No	No	Yes	No	Yes	No	Yes [6]	Yes
**Political considerations**	No	No	Yes	No	No	Yes [3]	No	No	No	Yes	No	Yes	No	Yes [6]	Yes
**Human rights**	No	No	No	No	No	No	No	No	Yes	No	No	No	No	Yes	Yes
**Other considerations**	Yes [7]	No	No	Yes [7]	Yes [7]	Yes [7]	No	No	No	Yes [7]	Yes [7]	Yes [7]	No	No	Yes [7]

*The map was constructed based on the criteria considered by the two most cited frameworks (GRADE EtD and WHO-INTEGRATE).
[1]As part of balance of benefits/harms
[2]As a sub-criteria in the "Balance of health benefits and harms"
[3]Partially yes
[4]As a subcriterion in "Human rights and sociocultural acceptability"
[5]Sustainability was identified as a criterion encompassing sub-criteria related to ecological, economic, and social considerations
[6]As a subcriterion in "Feasibility and health system considerations"
[7]Other considerations are: Evidence gaps (n=1); Stakeholder engagement (n=3); Transferability (n=1); Complementarities and interactions among strategies (n=1); Social impact (n=1); Implications for the course of the pandemic and its impact on health (n=1)

References

1. Moberg J, Oxman AD, Rosenbaum S, Schünemann HJ, Guyatt G, Flottorp S, et al. The GRADE Evidence to Decision (EtD) framework for health system and public health decisions. Health Res Policy Syst [Internet]. 2018 May 29;16(1):45. Available from: http://dx.doi.org/10.1186/s12961-018-0320-2

2. Alonso-Coello P, Schünemann HJ, Moberg J, Brignardello-Petersen R, Akl EA, Davoli M, et al. GRADE Evidence to Decision (EtD) frameworks: a systematic and transparent approach to making well informed healthcare choices. 1: Introduction. BMJ [Internet]. 2016 Jun 28;353:i2016. Available from: http://dx.doi.org/10.1136/bmj.i2016

3. Alonso-Coello P, Oxman AD, Moberg J, Brignardello-Petersen R, Akl EA, Davoli M, et al. GRADE Evidence to Decision (EtD) frameworks: a systematic and transparent approach to making well informed healthcare choices. 2: Clinical practice guidelines. BMJ [Internet]. 2016 Jun 30;353:i2089. Available from: http://dx.doi.org/10.1136/bmj.i2089

4. Andrews J, Guyatt G, Oxman AD, Alderson P, Dahm P, Falck-Ytter Y, et al. GRADE guidelines: 14. Going from evidence to recommendations: the significance and presentation of recommendations. J Clin Epidemiol [Internet]. 2013 Jul;66(7):719–25. Available from: http://dx.doi.org/10.1016/j.jclinepi.2012.03.013

5. Andrews JC, Schünemann HJ, Oxman AD, Pottie K, Meerpohl JJ, Coello PA, et al. GRADE guidelines: 15. Going from evidence to recommendation-determinants of a recommendation’s direction and strength. J Clin Epidemiol [Internet]. 2013 Jul;66(7):726–35. Available from: http://dx.doi.org/10.1016/j.jclinepi.2013.02.003

6. Schünemann HJ, Mustafa R, Brozek J, Santesso N, Alonso-Coello P, Guyatt G, et al.

GRADE Guidelines: 16. GRADE evidence to decision frameworks for tests in clinical practice and public health. J Clin Epidemiol [Internet]. 2016 Aug;76:89–98. Available from: http://dx.doi.org/10.1016/j.jclinepi.2016.01.032

7. Schünemann HJ, Wiercioch W, Brozek J, Etxeandia-Ikobaltzeta I, Mustafa RA, Manja V, et al. GRADE Evidence to Decision (EtD) frameworks for adoption, adaptation, and de novo development of trustworthy recommendations: GRADE-ADOLOPMENT. J Clin Epidemiol [Internet]. 2017 Jan;81:101–10. Available from: http://dx.doi.org/10.1016/j.jclinepi.2016.09.009

8. Piggott T, Brozek J, Nowak A, Dietl H, Dietl B, Saz-Parkinson Z, et al. Using GRADE evidence to decision frameworks to choose from multiple interventions. J Clin Epidemiol [Internet]. 2021 Feb;130:117–24. Available from: http://dx.doi.org/10.1016/j.jclinepi.2020.10.016

9. ACIP Evidence to Recommendation User’s Guide [Internet]. Advisory Committee on Immunization Practice (ACIP); 2020 Oct [cited 2023 Jun 2]. Available from: https://www.cdc.gov/vaccines/acip/recs/grade/downloads/acip-evidence-rec-frame-user-guide.pdf

10. Lee G, Carr W, ACIP Evidence-Based Recommendations Work Group. Updated Framework for Development of Evidence-Based Recommendations by the Advisory Committee on Immunization Practices. Morbidity and Mortality Weekly Report [Internet]. 2018 Nov 16 [cited 2023 Jun 2];67(45):1271–2. Available from: https://www.cdc.gov/mmwr/volumes/67/wr/pdfs/mm6745a4-H.pdf

11. Ahmed F. U.S. Advisory Committee on Immunization Practices (ACIP) Handbook for Developing Evidence-based Recommendations [Internet]. Centers for Disease Control and Prevention; 2013 Nov [cited 2023 Jun 2]. Available from: https://www.cdc.gov/vaccines/acip/recs/grade/downloads/handbook.pdf

12. Something went wrong [Internet]. [cited 2023 Jun 2]. Available from:

https://idsociety.sharepoint.com/sites/CAPG/Shared%20Documents/Forms/AllItems.aspx?id=%2Fsites%2FCAPG%2FShared%20Documents%2FPractice%20Guidelines%2FInternal%20Guideline%20Policies%20and%20Procedures%2FHandbook%2FIDSA%20Handbook%20for%20CPG%20Development%202021%2D01%2D19%2Epdf&parent=%2Fsites%2FCAPG%2FShared%20Documents%2FPractice%20Guidelines%2FInternal%20Guideline%20Policies%20and%20Procedures%2FHandbook&p=true&ga=1

13. Kroke A, Schmidt A, Amini AM, Kalotai N, Lehmann A, Haardt J, et al. Dietary protein intake and health-related outcomes: a methodological protocol for the evidence evaluation and the outline of an evidence to decision framework underlying the evidence-based guideline of the German Nutrition Society. Eur J Nutr [Internet]. 2022 Jun;61(4):2091–101. Available from: http://dx.doi.org/10.1007/s00394-021-02789-5

14. Guldbrandsson K, Stenström N, Winzer R. The DECIDE evidence to recommendation framework adapted to the public health field in Sweden. Health Promot Int [Internet]. 2016 Dec;31(4):749–54. Available from: http://dx.doi.org/10.1093/heapro/dav060

15. Barlam TF, Cosgrove SE, Abbo LM, MacDougall C, Schuetz AN, Septimus EJ, et al. Implementing an Antibiotic Stewardship Program: Guidelines by the Infectious Diseases Society of America and the Society for Healthcare Epidemiology of America. Clin Infect Dis [Internet]. 2016 May 15;62(10):e51–77. Available from: http://dx.doi.org/10.1093/cid/ciw118

16. Iversen BG, Vestrheim DF, Flottorp S, Denison E, Oxman AD. COVID-19-EPIDEMIC : Should individuals in the community without respiratory symptoms wear facemasks to reduce the spread of COVID-19?–a rapid review [Internet]. Norwegian Institute of Public Health; 2020. Available from: https://www.fhi.no/globalassets/dokumenterfiler/rapporter/2020/should-

individuals-in-the-community-without-respiratory-symptoms-wear-facemasks-to-reduce-the-spread-of-covid-19-report-2020.pdf

17. WHO Guidelines for malaria [Internet]. World Health Organization; 2023 Mar. Available from: https://www.who.int/publications/i/item/guidelines-for-malaria

18. de With K, Allerberger F, Amann S, Apfalter P, Brodt HR, Eckmanns T, et al. Strategies to enhance rational use of antibiotics in hospital: a guideline by the German Society for Infectious Diseases. Infection [Internet]. 2016 Jun;44(3):395–439. Available from: http://dx.doi.org/10.1007/s15010-016-0885-z

19. Rosenbaum SE, Moberg J, Glenton C, Schünemann HJ, Lewin S, Akl E, et al. Developing Evidence to Decision Frameworks and an Interactive Evidence to Decision Tool for Making and Using Decisions and Recommendations in Health Care. Glob Chall [Internet]. 2018 Sep;2(9):1700081. Available from: http://dx.doi.org/10.1002/gch2.201700081

20. Li SA, Alexander PE, Reljic T, Cuker A, Nieuwlaat R, Wiercioch W, et al. Evidence to Decision framework provides a structured "roadmap" for making GRADE guidelines recommendations. J Clin Epidemiol [Internet]. 2018 Dec;104:103–12. Available from: http://dx.doi.org/10.1016/j.jclinepi.2018.09.007

21. Neumann I, Brignardello-Petersen R, Wiercioch W, Carrasco-Labra A, Cuello C, Akl E, et al. The GRADE evidence-to-decision framework: a report of its testing and application in 15 international guideline panels. Implement Sci [Internet]. 2016 Jul 15;11:93. Available from: http://dx.doi.org/10.1186/s13012-016-0462-y

22. Meneses-Echavez JF, Rosenbaum S, Rada G, Flottorp S, Moberg J, Alonso-Coello P. Users' experiences with an interactive Evidence to Decision (iEtD) framework: a qualitative analysis. BMC Med Inform Decis Mak [Internet]. 2021 May 25;21(1):169. Available from: http://dx.doi.org/10.1186/s12911-021-01532-8

23. Friesen VM, Mbuya MNN, Wieringa FT, Nelson CN, Ojo M, Neufeld LM. Decisions to Start, Strengthen, and Sustain Food Fortification Programs: An Application of the Grading of Recommendations Assessment, Development, and Evaluation (GRADE) Evidence to Decision (EtD) Framework in Nigeria. Curr Dev Nutr [Internet]. 2022 Mar;6(3):nzac010. Available from: http://dx.doi.org/10.1093/cdn/nzac010

24. Stadelmaier J, Rehfuess EA, Forberger S, Eisele-Metzger A, Nagavci B, Schünemann HJ, et al. Using GRADE Evidence to Decision frameworks to support the process of health policy-making: an example application regarding taxation of sugar-sweetened beverages. Eur J Public Health [Internet]. 2022 Nov 28;32(Suppl 4):iv92–100. Available from: http://dx.doi.org/10.1093/eurpub/ckac077

25. Moleman M, Jerak-Zuiderent S, van de Bovenkamp H, Bal R, Zuiderent-Jerak T. Evidence-basing for quality improvement; bringing clinical practice guidelines closer to their promise of improving care practices. J Eval Clin Pract [Internet]. 2022 Dec;28(6):1003–26. Available from: http://dx.doi.org/10.1111/jep.13659

26. Stalteri Mastrangelo R, Santesso N, Bognanni A, Darzi A, Karam S, Piggott T, et al. Consideration of antimicrobial resistance and contextual factors in infectious disease guidelines: a systematic survey. BMJ Open [Internet]. 2021 Jul 30;11(7):e046097. Available from: http://dx.doi.org/10.1136/bmjopen-2020-046097

27. Rehfuess EA, Stratil JM, Scheel IB, Portela A, Norris SL, Baltussen R. The WHO-INTEGRATE evidence to decision framework version 1.0: integrating WHO norms and values and a complexity perspective. BMJ Glob Health [Internet]. 2019 Jan 25;4(Suppl 1):e000844. Available from: http://dx.doi.org/10.1136/bmjgh-2018-000844

28. Stratil JM, Baltussen R, Scheel I, Nacken A, Rehfuess EA. Development of the WHO-INTEGRATE evidence-to-decision framework: an overview of systematic reviews of decision criteria for health decision-making. Cost Eff Resour Alloc [Internet]. 2020

Feb 11;18:8. Available from: http://dx.doi.org/10.1186/s12962-020-0203-6

29. Guidelines on sanitation and health [Internet]. World Health Organization; 2018. Available from: https://www.who.int/publications/i/item/9789241514705

30. 027- ARN. S3-Guideline Measures for the prevention and control of SARS-CoV-2 transmission in schools | Living Guideline. AWMF; 2022 Sep.

31. Stratil JM, Paudel D, Setty KE, Menezes de Rezende CE, Monroe AA, Osuret J, et al. Advancing the WHO-INTEGRATE Framework as a Tool for Evidence-Informed, Deliberative Decision-Making Processes: Exploring the Views of Developers and Users of WHO Guidelines. Int J Health Policy Manag [Internet]. 2022 May 1;11(5):629–41. Available from: http://dx.doi.org/10.34172/ijhpm.2020.193

32. Murano M, Chou D, Costa ML, Turner T. Using the WHO-INTEGRATE evidence-to-decision framework to develop recommendations for induction of labour. Health Res Policy Syst [Internet]. 2022 Nov 7;20(1):125. Available from: http://dx.doi.org/10.1186/s12961-022-00901-7

33. Wabnitz K, Rueb M, Pfadenhauer LM, Strahwald B, Rehfuess EA. Rapid development of an evidence- and consensus-based guideline for controlling transmission of SARS-CoV-2 in schools during a public health emergency - A process evaluation. Front Public Health [Internet]. 2023 Mar 30;11:1075210. Available from: http://dx.doi.org/10.3389/fpubh.2023.1075210

34. Lyn R, Aytur S, Davis TA, Eyler AA, Evenson KR, Chriqui JF, et al. Policy, systems, and environmental approaches for obesity prevention: a framework to inform local and state action. J Public Health Manag Pract [Internet]. 2013 May-Jun;19(3 Suppl 1):S23–33. Available from: http://dx.doi.org/10.1097/PHH.0b013e3182841709

35. Spencer LM, Schooley MW, Anderson LA, Kochtitzky CS, DeGroff AS, Devlin HM, et al. Seeking best practices: a conceptual framework for planning and improving

evidence-based practices. Prev Chronic Dis [Internet]. 2013 Dec 12;10:E207. Available from: http://dx.doi.org/10.5888/pcd10.130186

36. Ismail SJ, Hardy K, Tunis MC, Young K, Sicard N, Quach C. A framework for the systematic consideration of ethics, equity, feasibility, and acceptability in vaccine program recommendations. Vaccine [Internet]. 2020 Aug 10;38(36):5861–76. Available from: http://dx.doi.org/10.1016/j.vaccine.2020.05.051

37. Ismail SJ, Langley JM, Harris TM, Warshawsky BF, Desai S, FarhangMehr M. Canada's National Advisory Committee on Immunization (NACI): evidence-based decision-making on vaccines and immunization. Vaccine [Internet]. 2010 Apr 19;28 Suppl 1:A58–63. Available from: http://dx.doi.org/10.1016/j.vaccine.2010.02.035

38. Ismail SJ, Tunis MC, Zhao L, Quach C. Navigating inequities: a roadmap out of the pandemic. BMJ Glob Health [Internet]. 2021 Jan;6(1). Available from: http://dx.doi.org/10.1136/bmjgh-2020-004087

39. An Advisory Committee Statement (ACS) National Advisory Committee on Immunization (NACI): Updated Recommendations on the Use of Herpes Zoster Vaccines [Internet]. Public Health Agency of Canada; 2018 Jun. Available from: https://www.canada.ca/content/dam/phac-aspc/documents/services/publications/healthy-living/updated-recommendations-use-herpes-zoster-vaccines-eng.pdf

40. An Advisory Committee Statement (ACS) National Advisory Committee on Immunization (NACI) - Guidance on COVID-19 vaccine booster doses: Initial considerations for 2023 [Internet]. Public Health Agency of Canada; 2023 Jan. Available from: https://www.canada.ca/content/dam/phac-aspc/documents/services/immunization/national-advisory-committee-on-immunization-naci/guidance-covid-19-vaccine-booster-doses-initial-considerations-2023/guidance-covid-19-vaccine-booster-doses-initial-considerations-2023.pdf

41. Rasul G. A Framework for Improving Policy Priorities in Managing COVID-19 Challenges in Developing Countries. Front Public Health [Internet]. 2020 Oct 14;8:589681. Available from: http://dx.doi.org/10.3389/fpubh.2020.589681

42. Stratil JM, Voss M, Arnold L. WICID framework version 1.0: criteria and considerations to guide evidence-informed decision-making on non-pharmacological interventions targeting COVID-19. BMJ Glob Health [Internet]. 2020 Nov;5(11). Available from: http://dx.doi.org/10.1136/bmjgh-2020-003699

43. Van 't Veer P, Grammatikaki E, Matthys C, Raats MM, Contor L. EURRECA-Framework for Aligning Micronutrient Recommendations. Crit Rev Food Sci Nutr [Internet]. 2013;53(10):988–98. Available from: http://dx.doi.org/10.1080/10408398.2012.742857

44. Dhonukshe-Rutten RAM, Timotijevic L, Cavelaars AEJM, Raats MM, de Wit LS, Doets EL, et al. European micronutrient recommendations aligned: a general framework developed by EURRECA. Eur J Clin Nutr [Internet]. 2010 Jun;64 Suppl 2:S2–10. Available from: http://dx.doi.org/10.1038/ejcn.2010.55

45. Krahn M, Miller F, Bayoumi A, Brooker AS, Wagner F, Winsor S, et al. DEVELOPMENT OF THE ONTARIO DECISION FRAMEWORK: A VALUES BASED FRAMEWORK FOR HEALTH TECHNOLOGY ASSESSMENT. Int J Technol Assess Health Care [Internet]. 2018 Jun;34(3):290–9. Available from: http://dx.doi.org/10.1017/S0266462318000235

46. Almeida ND, Mines L, Nicolau I, Sinclair A, Forero DF, Brophy JM, et al. A Framework for Aiding the Translation of Scientific Evidence into Policy: The Experience of a Hospital-Based Technology Assessment Unit. Int J Technol Assess Health Care [Internet]. 2019 Jan;35(3):204–11. Available from: http://dx.doi.org/10.1017/S0266462319000254

47. Veglia A, Pahwa M, Demers PA. Establishing a Policy Framework for the Primary

Prevention of Occupational Cancer: A Proposal Based on a Prospective Health Policy Analysis. Saf Health Work [Internet]. 2017 Mar;8(1):29–35. Available from: http://dx.doi.org/10.1016/j.shaw.2016.07.001

48. Votruba N, Grant J, Thornicroft G. The EVITA framework for evidence-based mental health policy agenda setting in low- and middle-income countries. Health Policy Plan [Internet]. 2020 May 1;35(4):424–39. Available from: http://dx.doi.org/10.1093/heapol/czz179

49. Votruba N, Grant J, Thornicroft G. EVITA 2.0, an updated framework for understanding evidence-based mental health policy agenda-setting: tested and informed by key informant interviews in a multilevel comparative case study. Health Res Policy Syst [Internet]. 2021 Mar 10;19(1):35. Available from: http://dx.doi.org/10.1186/s12961-020-00651-4

50. Shafaghat T, Bastani P, Nasab MHI, Bahrami MA, Montazer MRA, Zarchi MKR, et al. A framework of evidence-based decision-making in health system management: a best-fit framework synthesis. Arch Public Health [Internet]. 2022 Mar 29;80(1):96. Available from: http://dx.doi.org/10.1186/s13690-022-00843-0

51. Canfell OJ, Davidson K, Sullivan C, Eakin EE, Burton-Jones A. PREVIDE: A Qualitative Study to Develop a Decision-Making Framework (PREVention decIDE) for Noncommunicable Disease Prevention in Healthcare Organisations. Int J Environ Res Public Health [Internet]. 2022 Nov 18;19(22). Available from: http://dx.doi.org/10.3390/ijerph192215285

52. Guide to Community Preventive Services. Methods Manual for Community Guide Systematic Reviews [Internet]. [cited 2023 Jun 2]. Available from: https://www.thecommunityguide.org/pages/methods-manual.html

53. HIV Prevention and Control: Partner Services to Increase HIV Testing [Internet]. Community Preventive Services Task Force; 2021 Jul. Available from:

https://www.thecommunityguide.org/findings/hiv-prevention-partner-services-interventions-increase-hiv-testing.html

54. CPSTF Findings for Increasing Vaccination [Internet]. Community Preventive Services Task Force; 2016 Feb. Available from: https://www.thecommunityguide.org/pages/task-force-findings-increasing-vaccination.html

55. Guide to Community Preventive Services. Methods Manual for Community Guide Systematic Reviews [Internet]. [cited 2023 Jun 2]. Available from: https://www.thecommunityguide.org/pages/methods-manual.html
